# Inequality and heterogeneity in health-related quality of life: findings based on a large sample of cross-sectional EQ-5D-5L data from the Swedish general population

**DOI:** 10.1007/s11136-021-02982-3

**Published:** 2021-10-10

**Authors:** Fitsum Sebsibe Teni, Ulf-G. Gerdtham, Reiner Leidl, Martin Henriksson, Mimmi Åström, Sun Sun, Kristina Burström

**Affiliations:** 1 Health Outcomes and Economic Evaluation Research Group, Stockholm Centre for Healthcare Ethics, Department of Learning, Informatics, Management and Ethics, Karolinska Institutet, Tomtebodavägen 18a, 171 77 Stockholm, Sweden; 2 Department of Economics, Lund University, Lund, Sweden; 3 Health Economics Unit, Department of Clinical Sciences in Malmö, Lund University, Lund, Sweden; 4 Institute for Health Economics and Health Care Management, Helmholtz Zentrum München, German Research Center for Environmental Health, Neuherberg, Germany; 5 Munich Center of Health Sciences, Ludwig-Maximilians University, Munich, Germany; 6 Center for Medical Technology Assessment, Department of Health, Medicine and Caring Sciences, Linköping University, Linköping, Sweden; 7 Equity and Health Policy Research Group, Department of Global Public Health, Karolinska Institutet, Stockholm, Sweden; 8 Department of Epidemiology and Global Health, Umeå University, Umeå, Sweden

**Keywords:** EQ-5D-5L, General population, Inequalities in health status, Reference data, Socioeconomic group, Sweden

## Abstract

**Purpose:**

This study aimed to investigate inequality and heterogeneity in health-related quality of life (HRQoL) and to provide EQ-5D-5L population reference data for Sweden.

**Methods:**

Based on a large Swedish population-based survey, 25,867 respondents aged 30‒104 years, HRQoL is described by sex, age, education, income, economic activity, health-related behaviours, self-reported diseases and conditions. Results are presented by EQ-5D-5L dimensions, respondents rating of their overall health on the EQ visual analogue scale (EQ VAS), VAS index value and TTO (time trade-off) index value allowing for calculation of quality-adjusted life years (QALYs). Ordinary Least Squares and multivariable logistic regression analyses were used to study inequalities in observed EQ VAS score between socioeconomic groups and the likelihood to report problems on the dimensions, respectively, adjusted for confounders.

**Results:**

In total, 896 different health states were reported; 24.1% did not report any problems. Most problems were reported with pain/discomfort. Women reported worse HRQoL than men, and health deteriorated with age. The strongest association between diseases and conditions and EQ VAS score was seen for depression and mental health problems. There was a socioeconomic gradient in HRQoL; adjusting for health-related behaviours, diseases and conditions slightly reduced the differences between educational groups and income groups, but socioeconomic inequalities largely remained.

**Conclusion:**

EQ-5D-5L population reference (norms) data are now available for Sweden, including socioeconomic differentials. Results may be used for comparisons with disease-specific populations and in health economic evaluations. The observed socioeconomic inequality in HRQoL should be of great importance for policy makers concerned with equity aspects.

**Supplementary Information:**

The online version contains supplementary material available at 10.1007/s11136-021-02982-3.

## Introduction

Assessing population health status, and its distribution in subgroups, and exploring health inequalities associated with social determinants of health [1] is important in determining targets for and evaluating efficiency and equity aspects of health policy [2]. Health is a multi-dimensional concept and policy makers may need more specific information on which health dimensions are most affected in different groups in the population to better guide the investments in health policy. Although, a multi-dimensional instrument may be preferable for such purposes, it may, at the same time, be desirable to also convert the results into a single measure of health, weighing together the different dimensions .

The generic Health-Related Quality of Life (HRQoL) instrument EQ-5D-5L allows respondents to report their health in five dimension and five severity levels (yielding 3125 health states) and rate their overall health perception on the EQ visual analogue scale (EQ VAS) [3, 4]. This version was developed to increase discrimination between less severe health problems and to recognize small changes in health status, compared to the EQ-5D-3L with three severity levels, a welcomed improvement in granularity when used for economic evaluation as well as in population health status surveys (4). How problems are reported by the EQ-5D-5L descriptive system and how rating on EQ VAS differ by subgroups of the population reflect the distribution of health in the population. To assign a single index value for each health state, value sets can be applied using different valuation methods (e.g., time trade-off (TTO) and VAS valuations) and sources of valuations (based on own experience or on hypothetical states) [5–7]. Index values, or weights, the quality component corresponding to the 0‒1 scale (dead‒full health) required for calculation of Quality-Adjusted Life Years (QALYs) obtained through the EQ-5D-5L instrument can be used in economic evaluation [8].

Large sets of EQ-5D-5L data, commonly based on cross-sectional general population surveys, can serve as important population reference data, known also as norms data [9]. Population reference data for EQ-5D-5L can provide useful guidance regarding interpretation of results when the instrument is used in specific patient groups, for instance by comparing their results with data for the average person of the same sex and age in the general population [3, 9]. Subgroup reference data may be important for decision makers for example to understand the burden of disease in a specific patient population. They could also provide a reference to quantify the overall inequalities in health status [10]. Population reference data, with sex- and age-specific QALY-weights, are also important in any economic evaluation modelling long-term health outcomes of specific interventions.

Population reference data using the three-level severity version EQ-5D-3L [11, 12], as well as reference data for adolescents using the youth version EQ-5D-Y-3L [13] are available for Sweden. The EQ-5D-5L is increasingly used in Sweden – in health care and in quality registers which underlines the need of reference data also for the EQ-5D-5L [14]. However, until now, Sweden lacks large-scale EQ-5D-5L results for the general population stratified by subgroups.

Population reference data for the EQ-5D-5L are available for several countries at national or regional level. Recently, data have been presented for United States [15], Canada [16], Bulgaria [17], Russia [18], and Slovenia [19], adding to previous data for China [20], Germany [21–23], Indonesia [24], Ireland [25], South Australia [26], Spain [27, 28], Trinidad and Tobago [29], and Uruguay [30].

In Sweden, the EQ-5D-5L was included in the large population-based health survey to more than 56,000 individuals [31], enabling comprehensive and stratified analyses. These data were used for estimation of the Swedish experience-based value sets [32].

In the present study, we use these data to describe HRQoL in the population, by different subgroups and individual characteristics. The study investigates both systematic inequalities in HRQoL related to characteristics such as sex, age, education, income, and economic activity, and individual heterogeneity linked to medical and behavioural characteristics such as self-reported diseases and health conditions, and health-related behaviours. In addition, the paper provides EQ-5D-5L population reference data for Sweden.

## Materials and methods

### Study population

Data were obtained from the population-based health survey Life & Health 2017, a cross-sectional self-administered postal survey in the regions/county councils in mid-Sweden (CDUST Region) with a 47.1% response rate [31] (this report does not include the EQ-5D-5L results). These data were used in the publication presenting the Swedish EQ-5D-5L value sets, see further details [32]. Internal response rates, information on non-respondents, categorisation of demographic and socioeconomic information and variables for health-related behaviours, diseases diagnosed by a physician and self-reported conditions are described in the Online Resource. Survey data were linked to Statistics Sweden’s Longitudinal Integrated Database for Health Insurance and Labour Market Studies to obtain sociodemographic characteristics for each respondent [33]. The sociodemographic composition of the CDUST Region is similar to the overall census data from Sweden (Online Resource Table S1) [32]. The survey has been approved by the Regional Ethical Review Board in Uppsala (Dnr: 2015/417). The present study has been approved by the Swedish Ethical Review Authority (Dnr: 2019–00763).

The EQ-5D-5L was included in the survey to persons aged 30 years and above. The respondents classified their own health into five dimensions (mobility; self-care; usual activities; pain/discomfort; anxiety/depression) with five severity levels (no; slight; moderate; severe; extreme/unable) and rated their own overall health on the EQ VAS between 100 (best imaginable health) and 0 (worst imaginable health), yielding an observed EQ VAS score. To assign a single index value for each of the 3125 health states [4], the EQ-5D-5L value sets for Sweden based on TTO and VAS valuations were employed [32].

### Statistical analysis

Descriptive statistics were employed in calculation of the proportion of participants reporting no, slight, moderate, severe and extreme problems on the EQ-5D-5L dimensions, mean and median observed EQ VAS score, and mean TTO and VAS index values. Standard Deviation (SD) was reported for mean values; interquartile range (IQR) for median values. The chi-square test was used to test for statistically significant differences between groups in the proportion of any reported problems (slight, moderate, severe and extreme problems were collapsed into any problems). Independent t-test was used for comparison of mean VAS scores, mean TTO and VAS index values. We employed information on sex, age, education, income, and economic activity (information available for those up to 64 years) in the analyses related to inequality. In the analyses related to heterogeneity, information on self-reported diseases and health conditions, and health-related behaviours was used. SAS software 9.4 was used; 5% significance level was employed.

Ordinary Least Squares (OLS) regression, with robust Standard Errors (SE), was applied to study inequalities in mean observed EQ VAS score between socioeconomic groups while adjusting for sex, age and other factors entered as dummy variables [34]. The first model included education, and the second instead included income. We included education and income simultaneously in the third model because education can be seen as a factor underlying the association between income and health, i.e., education is generally defined in early life, and income is partly the outcome of educational achievements. In addition, by including both education and income in the same model we can also explore which was the stronger variable that might provide information about the relative strength of mechanisms. For respondents aged up to 64 years, additionally economic activity was included.

Further, separate models were assessed adjusting for health-related behaviours, diseases diagnosed by a physician, self-reported conditions, and BMI levels in addition to education and income. These analyses yield estimates, for instance, showing the association between health-related behaviours and mean EQ VAS score only adjusted for sex and age, but also the association when adjusting for socioeconomic group. The difference between these estimates may reflect to what extent differences between socioeconomic groups are explained by differences in health-related behaviours.

Multivariable logistic regression analysis was applied for estimations of Odds Ratios (OR) to investigate the relationship between the likelihood to report any problems on the five EQ-5D-5L dimensions and respondent’s sex, age, education, income, and economic activity.

## Results

Data from 25,867 individuals were used in the present study. The mean age was 64.3 years, 52.6% were women, and 42.5% were diagnosed with at least one disease (Online Resource Table S2). In total, 896 health states (28.7% of the 3125 possible states) were reported; 174 by ten or more, and 291 by five or more respondents (Online Resource Table S3). No problems on any dimension (state 11111) was reported by 24.1%; extreme problems on all dimensions (state 55555) was reported by six respondents (0.02%).

### Descriptive analysis across subgroups

#### HRQoL by sex and age groups

A general age gradient was observed, with deteriorating health status with age (results for the total sample in Online Resource Table S4), except among men (Table 1 showing only men) in the youngest age groups for all dimensions, and among women (Table 2 showing only women) in the youngest age groups in the self-care, usual activities, and anxiety/depression dimensions. In general, women reported worse health than men. In the anxiety/depression dimension, there was a bell-shaped age gradient, with the lowest proportion of reporting any problems among both men and women aged 65–69 years, more pronounced among women. Among men, the proportion reporting any problems was similar in the youngest and the oldest age groups. The highest mean EQ VAS score among men, 81.5, was found in the youngest age group. The highest mean TTO index value (0.938) as well as the mean VAS index value (82.0) were found in the age group 35‒39 years. Among women, the highest mean EQ VAS score, 80.0, was in the age group 64‒69 years, in which also the mean TTO index value was high (0.909) similar to the age group 35‒39 where the highest mean TTO index value (0.910) and the highest mean VAS index value (78.1) were found.

**Table 1:** Problems in EQ-5D-5L dimensions (%), mean and median EQ VAS score, mean TTO index value, and mean VAS index value, men, by age group (*n* = 12,249)

EQ-5D-5L dimension	Men by age group (years)														
	Total	30–34	35–39	40–44	45–49	50–54	55–59	60–64	65–69	70–74	75–79	80–84	85–89	90–94	95–104
	*n* = 12,249	*n* = 437	*n* = 501	*n* = 620	*n* = 750	*n* = 779	*n* = 842	*n* = 976	*n* = 1242	*n* = 2323	*n* = 1495	*n* = 842	*n* = 1014	*n* = 364	*n* = 64
Mobility															
No	68.0	91.5	93.0	90.0	86.0	82.8	78.2	74.5	74.0	68.5	60.0	47.0	32.5	23.6	14.1
Slight	16.3	6.2	6.0	6.9	9.9	10.6	14.1	15.3	15.4	17.4	19.9	24.5	26.7	25.3	21.9
Moderate	9.8	1.8	1.0	1.8	2.8	4.6	5.3	7.3	6.6	9.5	12.8	18.0	23.6	29.7	18.8
Severe	4.7	0.5	0.0	1.0	1.1	1.8	2.3	2.8	3.4	3.8	6.2	8.3	13.1	16.5	28.1
Extreme	1.1	0.0	0.0	0.3	0.3	0.1	0.1	0.2	0.6	0.9	1.1	2.1	4.0	5.0	17.2
Self-care															
No	88.4	97.2	99.0	96.4	96.3	93.3	92.8	92.0	92.5	89.9	86.6	79.7	71.8	60.4	46.9
Slight	6.7	2.1	1.0	2.1	2.4	5.1	5.2	5.9	4.4	7.0	8.5	12.0	12.2	15.4	10.9
Moderate	2.8	0.7	0.0	0.8	0.9	1.5	1.5	1.6	1.9	1.8	2.9	4.2	9.4	12.6	9.4
Severe	1.2	0.0	0.0	0.3	0.3	0.0	0.4	0.3	1.0	0.7	1.1	2.3	3.9	6.3	15.6
Extreme	0.9	0.0	0.0	0.3	0.1	0.0	0.1	0.1	0.1	0.6	0.9	1.9	2.7	5.2	17.2
Usual activities															
No	70.6	84.9	87.6	86.3	82.5	79.8	77.1	74.5	75.6	75.4	64.6	54.0	44.0	33.0	23.4
Slight	17.2	10.3	9.6	10.3	12.7	13.0	14.8	17.8	16.5	15.0	21.3	25.1	25.8	27.2	23.4
Moderate	6.7	2.3	2.2	2.1	2.5	3.7	4.5	4.5	4.4	5.9	8.4	12.6	15.9	17.6	15.6
Severe	3.4	1.8	0.4	1.1	1.6	2.4	2.5	2.5	3.2	2.4	4.0	4.4	8.5	11.5	7.8
Extreme	2.0	0.7	0.2	0.2	0.7	1.0	1.1	0.7	0.3	1.2	1.8	3.9	5.8	10.7	29.7
Pain/discomfort															
No	35.5	55.4	55.7	46.6	45.7	40.4	36.3	33.2	34.2	35.6	31.1	26.6	22.4	18.7	21.9
Slight	39.6	34.1	37.1	40.8	35.6	41.1	41.3	41.2	41.9	40.2	40.1	39.1	37.3	38.7	34.4
Moderate	20.2	8.2	6.0	10.5	15.6	13.0	17.0	21.7	19.7	19.8	23.9	27.6	32.2	34.6	31.2
Severe	4.4	2.3	1.2	1.9	2.3	5.0	4.8	3.6	4.0	4.0	4.6	6.3	7.6	7.4	12.5
Extreme	0.4	0.0	0.0	0.2	0.8	0.5	0.6	0.3	0.2	0.4	0.4	0.5	0.6	0.6	0.0
Anxiety/depression															
No	68.4	57.0	62.3	62.9	66.9	62.6	68.9	71.3	76.9	72.1	72.2	69.2	61.1	58.5	56.2
Slight	24.8	31.6	29.3	27.9	25.6	29.0	22.6	23.0	18.8	23.6	22.6	23.9	29.2	32.7	29.7
Moderate	4.9	7.3	7.0	7.1	5.2	5.8	5.7	3.5	3.5	3.1	4.4	4.5	7.1	6.9	9.4
Severe	1.6	3.0	1.2	1.6	1.7	2.2	2.4	1.7	0.6	1.1	0.7	2.1	2.3	1.9	3.1
Extreme	0.3	1.1	0.2	0.5	0.5	0.4	0.5	0.5	0.3	0.2	0.1	0.2	0.3	0.0	1.6
EQ VAS score (mean) [SD]	76.6 [18.3]	81.5 [14.3]	80.6 [14.0]	80.4 [15.1]	80.0 [15.9]	79.2 [16.7]	79.0 [17.3]	78.6 [16.7]	79.5 [16.9]	78.4 [17.2]	75.0 [18.3]	70.8 [19.7]	65.2 [21.2]	63.5 [21.9]	52.8 [25.4]
EQ VAS score (median) [IQR]	80.0 [20.0]	85.0 [15.0]	83.0 [15.0]	85.0 [15.0]	85.0 [15.0]	82.0 [20.0]	84.0 [15.0]	80.0 [18.0]	85.0 [15.0]	80.0 [20.0]	80.0 [25.0]	75.0 [25.0]	70.0 [30.0]	70.0 [30.0]	52.5 [45.0]
TTO index value (mean) [SD]	0.898 [0.112]	0.925 [0.088]	0.938 [0.061]	0.930 [0.080]	0.924 [0.093]	0.914 [0.103]	0.910 [0.108]	0.910 [0.099]	0.915 [0.096]	0.909 [0.100]	0.892 [0.109]	0.865 [0.127]	0.831 [0.142]	0.803 [0.143]	0.751 [0.166]
VAS index value (mean) [SD]	76.8 [13.9]	80.3 [11.1]	82.0 [8.6]	80.8 [10.4]	80.2 [11.5]	79.0 [12.6]	78.5 [13.1]	78.3 [12.3]	78.9 [12.2]	78.0 [12.8]	75.8 [13.8]	72.5 [15.5]	68.3 [16.7]	64.9 [16.9]	58.2 [19.9]

**Table 2:** Problems in EQ-5D-5L dimensions (%), mean and median EQ VAS score, mean TTO index value, and mean VAS index value, women, by age group (*n* = 13,618)

EQ-5D-5L dimension	Women by age group (years)														
	Total	30–34	35–39	40–44	45–49	50–54	55–59	60–64	65–69	70–74	75–79	80–84	85–89	90–94	95–104
	*n* = 13,618	*n* = 690	*n* = 701	*n* = 853	*n* = 972	*n* = 1045	*n* = 1075	*n* = 1168	*n* = 1405	*n* = 2166	*n* = 1477	*n* = 881	*n* = 748	*n* = 339	*n* = 98
Mobility															
No	67.3	87.5	87.3	86.0	83.3	78.1	74.8	71.8	70.3	66.3	56.0	44.4	30.1	19.8	10.2
Slight	18.0	9.1	9.4	9.7	12.1	13.1	14.1	18.8	19.2	19.7	24.7	25.9	29.7	24.5	17.4
Moderate	9.5	2.5	2.1	3.3	3.3	6.4	7.6	6.6	7.0	9.9	13.5	19.1	23.7	29.5	23.5
Severe	4.0	0.6	1.0	0.9	1.0	2.2	3.2	2.2	3.1	3.0	5.2	8.4	12.6	17.1	25.5
Extreme	1.2	0.3	0.1	0.0	0.2	0.2	0.3	0.6	0.5	1.1	0.7	2.3	4.0	9.1	23.5
Self-care															
No	89.9	95.8	97.9	96.0	95.2	93.5	91.6	93.3	93.7	92.4	89.4	82.3	73.1	50.2	25.5
Slight	5.4	3.5	1.6	2.9	3.6	4.6	6.1	3.9	4.6	4.5	6.1	8.8	10.0	16.8	18.4
Moderate	2.8	0.7	0.4	0.6	1.0	1.5	1.8	1.5	1.2	2.0	3.0	5.6	8.7	18.0	18.4
Severe	1.1	0.0	0.0	0.5	0.2	0.4	0.4	0.8	0.4	0.6	1.0	2.0	4.6	6.8	16.3
Extreme	0.8	0.0	0.1	0.0	0.0	0.0	0.1	0.4	0.1	0.6	0.4	1.2	3.6	8.3	21.4
Usual activities															
No	67.9	78.7	79.6	77.5	76.0	74.6	70.8	70.5	75.1	71.3	64.2	51.6	37.7	23.9	16.3
Slight	19.0	13.6	12.3	13.4	15.8	14.6	18.7	19.9	17.8	19.3	22.5	26.9	30.0	22.4	21.4
Moderate	7.4	5.1	4.8	5.7	4.9	6.2	5.5	5.7	4.4	6.2	8.6	13.2	16.7	23.6	11.2
Severe	3.7	2.0	2.8	2.7	2.7	3.5	4.2	3.4	1.8	1.8	3.2	5.1	9.0	15.9	22.4
Extreme	1.9	0.6	0.4	0.7	0.5	1.0	0.8	0.5	0.8	1.4	1.4	3.2	6.7	14.2	28.6
Pain/discomfort															
No	28.8	45.2	44.6	39.4	36.1	30.3	27.5	27.3	26.5	28.4	22.6	19.0	16.8	14.2	11.2
Slight	40.1	38.3	37.8	40.7	39.5	40.4	40.9	42.9	44.1	40.4	41.6	37.6	34.6	32.4	30.6
Moderate	24.7	12.5	14.1	15.2	18.3	21.9	23.7	23.5	25.4	25.4	29.4	34.7	37.3	40.1	44.9
Severe	6.0	3.6	3.1	4.6	5.4	6.6	7.2	6.0	4.0	5.5	6.0	8.1	10.0	12.4	10.2
Extreme	0.5	0.4	0.3	0.1	0.6	0.8	0.6	0.3	0.1	0.4	0.3	0.7	1.2	0.9	3.1
Anxiety/depression															
No	57.8	43.0	48.5	49.8	53.8	54.4	60.7	63.9	67.0	65.0	59.3	57.0	51.6	50.2	35.7
Slight	32.4	38.1	38.4	36.8	34.8	33.9	29.2	28.7	27.8	28.3	33.3	32.8	36.6	37.2	37.8
Moderate	7.0	12.5	8.6	9.5	7.6	7.9	7.2	5.1	3.9	4.9	6.0	7.5	8.8	8.0	20.4
Severe	2.4	4.9	3.6	3.0	3.1	3.2	2.5	1.9	1.2	1.5	1.0	2.4	2.1	4.7	6.1
Extreme	0.5	1.4	1.0	0.8	0.7	0.5	0.4	0.4	0.1	0.2	0.5	0.3	0.8	0.0	0.0
EQ VAS score (mean) [SD]	75.7 [19.0]	77.4 [16.8]	77.1 [17.1]	77.0 [17.6]	76.7 [17.8]	76.4 [18.5]	77.3 [18.9]	78.9 [17.4]	80.0 [16.9]	78.2 [18.3]	74.0 [18.6]	69.1 [21.2]	64.4 [21.1]	56.1 [22.8]	56.7 [23.2]
EQ VAS score (median) [IQR]	80.0 [20.0]	80.0 [20.0]	80.0 [20.0]	80.0 [20.0]	80.0 [20.0]	80.0 [20.0]	80.0 [20.0]	80.0 [20.0]	85.0 [22.0]	80.0 [20.0]	80.0 [25.0]	75.0 [30.0]	70.0 [30.0]	55.0 [35.0]	55.0 [35.0]
TTO index value (mean) [SD]	0.886 [0.116]	0.902 [0.105]	0.910 [0.098]	0.906 [0.100]	0.904 [0.104]	0.895 [0.113]	0.894 [0.112]	0.899 [0.106]	0.909 [0.092]	0.899 [0.105]	0.883 [0.110]	0.852 [0.132]	0.814 [0.144]	0.761 [0.151]	0.696 [0.161]
VAS index value (mean) [SD]	75.0 [14.2]	77.2 [12.8]	78.1 [12.1]	77.6 [12.4]	77.4 [12.6]	76.2 [13.6]	76.0 [13.6]	76.7 [13.0]	77.6 [11.8]	76.5 [13.1]	74.2 [13.6]	70.6 [15.6]	66.1 [16.8]	60.2 [17.2]	52.2 [17.9]

#### HRQoL by education, income and economic activity

Overall, respondents with low education reported more problems in the EQ-5D-5L dimensions and lower mean EQ VAS scores, TTO and VAS index values than those with high education (Table 3). However, this was less pronounced for the anxiety/depression dimension. In general, there was an income gradient in health status with those with lower income reporting more problems (Table 4). Exceptions were found for some dimensions where those in the first quintile (lowest) reported less problems than those in the second quintile. Those unemployed reported more problems in all dimensions and had lower mean EQ VAS scores, TTO and VAS index values than those employed (Online Resource Table S5). Comparing mean EQ VAS scores to mean VAS index values, low education signals additional burden via EQ VAS across all three groups observed which points at multiple-burden of low education: more problems reported, in consequence lower VAS index, and even lower self-reported EQ VAS score. The same pattern was seen for income quintiles.

**Table 3:** Problems in EQ-5D-5L dimensions (%), mean and median EQ VAS score, mean TTO index value, and mean VAS index value, by sex, 30‒104 years, by educational level^a^ (*n* = 25,726)

EQ-5D-5L dimension	Total sample (%)			Men (%)			Women (%)		
	Educational level			Educational level			Educational level		
	Low	Medium	High	Low	Medium	High	Low	Medium	High
	*n* = 6005	*n* = 11,027	*n* = 8694	*n* = 3218	*n* = 5268	*n* = 3691	*n* = 2787	*n* = 5759	*n* = 5003
Mobility									
No	52.1	68.1	78.0	53.9	69.0	79.0	50.0	67.4	77.2
Slight	22.6	17.7	12.6	22.0	16.6	10.8	23.3	18.7	14.0
Moderate	15.1	9.3	6.4	14.4	9.2	6.6	15.9	9.3	6.2
Severe	7.8	4.0	2.4	7.5	4.3	2.9	8.1	3.7	2.1
Extreme	2.4	0.9	0.6	2.2	0.9	0.6	2.7	1.0	0.5
Self-care									
No	81.6	89.9	93.8	82.1	89.0	93.2	81.0	90.8	94.3
Slight	8.7	6.0	4.0	9.2	6.7	4.4	8.1	5.4	3.8
Moderate	5.4	2.4	1.4	4.8	2.5	1.6	6.2	2.3	1.2
Severe	2.3	1.0	0.5	2.0	1.2	0.5	2.6	0.9	0.4
Extreme	1.9	0.7	0.3	1.8	0.6	0.3	2.1	0.7	0.3
Usual activities									
No	58.0	68.7	77.9	60.1	70.6	80.1	55.5	67.0	76.2
Slight	22.1	19.4	13.8	21.4	18.2	12.0	22.8	20.5	15.1
Moderate	10.2	6.8	5.2	9.4	6.3	4.9	11.2	7.3	5.4
Severe	5.7	3.4	2.3	5.2	3.2	2.2	6.2	3.5	2.4
Extreme	4.1	1.7	0.8	3.9	1.7	0.8	4.3	1.7	0.8
Pain/discomfort									
No	24.7	29.3	40.4	28.2	32.8	45.6	20.6	26.0	36.5
Slight	38.2	40.6	40.1	38.1	41.2	38.7	38.2	40.2	41.2
Moderate	29.1	24.0	16.0	27.4	20.8	12.8	31.1	26.9	18.4
Severe	7.2	5.7	3.3	5.7	4.8	2.7	9.1	6.5	3.7
Extreme	0.8	0.4	0.2	0.6	0.4	0.2	1.0	0.4	0.2
Anxiety/depression									
No	62.1	62.9	63.4	67.4	68.2	69.7	55.9	58.0	58.8
Slight	29.3	28.9	28.3	25.8	24.9	23.9	33.3	32.6	31.6
Moderate	6.0	5.7	6.2	4.8	5.0	4.8	7.5	6.4	7.2
Severe	2.1	2.1	1.7	1.7	1.5	1.4	2.6	2.6	1.9
Extreme	0.5	0.4	0.4	0.3	0.4	0.3	0.6	0.4	0.4
EQ VAS score (mean) [SD]	71.6 [20.5]	76.2 [18.8]	79.0 [16.6]	72.7 [19.7]	76.8 [18.2]	79.5 [16.4]	70.2 [21.3]	75.6 [19.2]	78.7 [16.7]
EQ VAS score (median) [IQR]	75.0 [30.0]	80.0 [20.0]	80.0 [17.0]	78.0 [30.0]	80.0 [20.0]	85.0 [15.0]	75.0 [35.0]	80.0 [25.0]	80.0 [20.0]
TTO index value (mean) [SD]	0.863 [0.132]	0.892 [0.113]	0.913 [0.096]	0.873 [0.126]	0.899 [0.110]	0.920 [0.093]	0.851 [0.138]	0.886 [0.115]	0.908 [0.097]
VAS index value (mean) [SD]	72.2 [15.9]	75.8 [13.8]	78.6 [12.1]	73.5 [15.4]	76.8 [13.7]	79.7 [11.9]	70.7 [16.3]	74.9 [13.9]	77.8 [12.3]

Educational levels: low= elementary school 9‒10 years; medium= secondary school 3‒4 years; high= more than 3‒4 years secondary school

**Table 4:** Problems in EQ-5D-5L dimensions (%), mean and median EQ VAS score, mean TTO index value, and mean VAS index value, by sex, 30‒104 years, by income quintile (*n* = 25,797)

EQ-5D-5L dimension	Total sample (%)					Men (%)					Women (%)				
	Income quintile					Income quintile					Income quintile				
	First (lowest)	Second	Third	Fourth	Fifth (highest)	First (lowest)	Second	Third	Fourth	Fifth (highest)	First (lowest)	Second	Third	Fourth	Fifth (highest)
	*n* = 5159	*n* = 5160	*n* = 5159	*n* = 5160	*n* = 5159	*n* = 1563	*n* = 2515	*n* = 2379	*n* = 2479	*n* = 3279	*n* = 3596	*n* = 2645	*n* = 2780	*n* = 2681	*n* = 1880
Mobility															
No	54.7	51.8	68.6	79.5	83.4	55.0	50.1	64.4	76.8	83.7	54.6	53.5	72.2	82.0	82.8
Slight	21.5	22.5	17.5	13.9	10.5	19.5	22.2	18.3	15.1	9.9	22.4	22.9	16.9	12.7	11.6
Moderate	14.0	16.1	9.6	4.7	4.0	13.4	17.6	11.2	5.7	4.2	14.3	14.6	8.2	3.7	3.7
Severe	7.6	7.5	3.4	1.6	1.6	9.0	8.2	4.9	2.1	1.9	7.0	6.8	2.1	1.3	1.2
Extreme	2.1	2.1	0.9	0.3	0.4	3.1	1.9	1.2	0.3	0.3	1.7	2.3	0.6	0.4	0.6
Self-care															
No	82.7	82.1	90.4	94.7	96.0	79.1	80.6	87.8	93.2	95.6	84.3	83.5	92.7	96.1	96.9
Slight	8.3	9.0	6.1	4.0	2.8	10.1	10.2	7.2	5.2	3.2	7.4	7.8	5.1	2.9	2.0
Moderate	5.0	5.6	1.9	0.8	0.6	5.3	5.8	2.8	1.2	0.6	4.9	5.4	1.2	0.5	0.5
Severe	2.4	1.8	0.9	0.3	0.4	3.2	1.8	1.3	0.4	0.4	2.0	1.9	0.6	0.2	0.3
Extreme	1.7	1.5	0.7	0.2	0.2	2.3	1.5	0.9	0.1	0.2	1.4	1.4	0.5	0.3	0.3
Usual activities															
No	57.0	57.0	69.7	79.1	83.1	57.3	56.6	68.1	78.3	83.7	56.9	57.4	71.0	79.8	82.0
Slight	22.6	23.2	18.8	14.5	11.9	20.9	23.1	19.0	15.2	11.2	23.3	23.3	18.5	13.9	13.0
Moderate	10.7	10.9	7.1	4.1	2.8	10.2	11.2	7.5	4.3	2.9	10.8	10.6	6.6	3.9	2.8
Severe	5.7	5.7	3.2	1.9	1.5	6.0	5.8	3.6	1.9	1.5	5.5	5.6	2.8	1.9	1.4
Extreme	4.1	3.2	1.3	0.4	0.7	5.6	3.4	1.7	0.3	0.7	3.5	3.1	1.0	0.5	0.8
Pain/discomfort															
No	24.3	23.6	31.2	36.9	43.5	30.4	24.6	34.0	38.4	44.8	21.6	22.5	28.7	35.4	41.3
Slight	37.6	39.0	40.0	42.6	40.2	35.6	40.1	38.6	41.8	40.2	38.4	38.0	41.2	43.4	40.2
Moderate	28.6	29.6	23.4	17.2	14.0	24.8	28.4	22.2	17.0	12.8	30.3	30.8	24.4	17.5	16.1
Severe	8.6	7.2	5.1	3.1	2.2	8.2	6.3	4.8	2.6	2.1	8.8	8.0	5.4	3.5	2.4
Extreme	0.9	0.7	0.3	0.2	0.1	1.0	0.6	0.4	0.2	0.1	0.8	0.8	0.3	0.2	0.0
Anxiety/depression															
No	54.5	59.4	62.7	65.7	71.6	58.9	64.4	68.0	71.6	73.7	52.6	54.7	58.2	60.2	68.0
Slight	33.1	30.8	28.9	27.7	23.7	28.0	27.5	25.0	23.4	22.5	35.3	34.0	32.2	31.7	25.8
Moderate	8.7	6.2	6.3	5.0	3.6	9.3	5.1	5.3	3.9	3.0	8.4	7.3	7.3	6.1	4.7
Severe	3.0	2.9	1.7	1.3	0.9	3.0	2.5	1.4	0.9	0.7	3.0	3.3	1.9	1.7	1.3
Extreme	0.7	0.6	0.4	0.3	0.1	0.8	0.5	0.3	0.2	0.1	0.7	0.7	0.4	0.3	0.2
EQ VAS score (mean) [SD]	70.7 [21.2]	71.4 [20.4]	76.8 [18.1]	79.8 [15.8]	81.2 [14.8]	70.4 [22.0]	70.9 [20.0]	76.0 [18.8]	79.8 [15.4]	81.4 [14.3]	70.8 [20.8]	71.8 [20.8]	77.6 [17.5]	79.8 [16.2]	81.0 [15.7]
EQ VAS score (median) [IQR]	75.0 [30.0]	75.0 [30.0]	80.0 [20.0]	81.0 [15.0]	85.0 [15.0]	75.0 [33.0]	75.0 [25.0]	80.0 [20.0]	81.0 [15.0]	85.0 [15.0]	75.0 [30.0]	75.0 [30.0]	80.0 [20.0]	82.0 [17.0]	85.0 [15.0]
TTO index value (mean) [SD]	0.856 [0.136]	0.861 [0.133]	0.895 [0.107]	0.918 [0.086]	0.929 [0.078]	0.855 [0.143]	0.864 [0.130]	0.894 [0.112]	0.920 [0.085]	0.931 [0.077]	0.857 [0.133]	0.858 [0.135]	0.897 [0.103]	0.916 [0.087]	0.925 [0.078]
VAS index value (mean) [SD]	71.4 [16.2]	71.9 [15.7]	76.2 [13.4]	79.1 [11.1]	80.7 [10.3]	71.5 [17.2]	72.3 [15.6]	76.2 [13.9]	79.5 [11.0]	81.1 [10.3]	71.3 [15.7]	71.6 [15.8]	76.2 [12.9]	78.8 [11.2]	80.1 [10.4]

^a^Educational levels: low= elementary school 9‒10 years; medium= secondary school 3‒4 years; high= more than 3‒4 years secondary school

#### HRQoL by disease, self-reported conditions, and self-rated health (SRH) and likelihood to report problems on EQ-5D-5L dimensions

The distribution of problems on dimensions, mean and median EQ VAS score, mean TTO and VAS index values are presented in the Online Resource for diseases diagnosed by a physician and self-reported conditions (Table S6), BMI groups (Table S7), self-reported stress, self-reported sickness, and number of diagnosed diseases (Table S8). The distribution of response options to the self-rated health (SRH) question (five response options on a Likert scale to the question ‘How is your general health today?’), by sex and age group, is presented in Table S9. Results showed that HRQoL was consistently related to the severity levels of respondents’ answers to the SRH question (Table S10). Online Resource shows the associations, presented as OR, between reporting any problems on the five EQ-5D-5L dimensions and respondent’s sex, age, education, income and economic activity (Tables S11 and S12).

### Inequality and heterogeneity analysis, results adjusted for confounders

#### Inequalities in EQ VAS score between socioeconomic groups

The association between education and income and mean EQ VAS score, adjusted for sex and age, is presented in Table 5. Mean EQ VAS score was significantly higher for those with medium and high education compared to those with low education (Model 1). When instead adjusting for income, there was a statistically significant income gradient in mean EQ VAS score (Model 2). When adjusting for both education and income, the differences between education and income groups remained, but the coefficients were reduced (Model 3). Women had a statistically lower mean EQ VAS score than men when adjusting for education (Model 1), but not when including income in the models.

**Table 5:** Ordinary Least Square (OLS) regression on mean EQ VAS score adjusted for sex, age, educational level and income, 30‒104 years (*n* = 23,899)

Variable	EQ VAS score								
	Model 1			Model 2			Model 3		
	Estimate	RSE	*P*-value	Estimate	RSE	*P*-value	Estimate	RSE	*P*-value
Intercept	**76.796**	**0.587**	** < .0001**	**73.988**	**0.611**	** < .0001**	**72.790**	**0.657**	** < .0001**
Sex^a^									
Women	** − 1.701**	**0.233**	** < .0001**	− 0.054	0.242	0.8243	− 0.281	0.246	0.2529
Age group^b^									
35–39	− 0.448	0.671	0.5050	− 1.094	0.675	0.1052	− 1.049	0.673	0.1192
40–44	− 0.499	0.655	0.4460	** − 2.116**	**0.657**	**0.0013**	** − 1.935**	**0.656**	**0.0032**
45–49	− 0.687	0.641	0.2840	** − 2.439**	**0.641**	**0.0001**	** − 2.180**	**0.642**	**0.0007**
50–54	− 0.982	0.646	0.1288	** − 2.756**	**0.642**	** < .0001**	** − 2.396**	**0.645**	**0.0002**
55–59	− 0.491	0.646	0.4474	** − 2.289**	**0.642**	**0.0004**	** − 1.883**	**0.643**	**0.0034**
60–64	0.420	0.620	0.4982	− 1.125	0.615	0.0673	− 0.655	0.618	0.2896
65–69	**1.566**	**0.596**	**0.0086**	0.794	0.591	0.1793	**1.272**	**0.594**	**0.0323**
70–74	0.339	0.563	0.5478	0.801	0.563	0.1547	**1.330**	**0.567**	**0.0190**
75–79	** − 3.243**	**0.609**	** < .0001**	** − 1.986**	**0.617**	**0.0013**	** − 1.444**	**0.621**	**0.0202**
80–84	** − 7.521**	**0.720**	** < .0001**	** − 6.143**	**0.725**	** < .0001**	** − 5.475**	**0.731**	** < .0001**
85–89	** − 12.551**	**0.748**	** < .0001**	** − 11.173**	**0.753**	** < .0001**	** − 10.416**	**0.762**	** < .0001**
90–94	** − 17.133**	**1.071**	** < .0001**	** − 15.727**	**1.068**	** < .0001**	** − 14.908**	**1.079**	** < .0001**
95–104	** − 21.650**	**2.144**	** < .0001**	** − 20.374**	**2.136**	** < .0001**	** − 19.445**	**2.147**	** < .0001**
Educational level^c, e^									
Medium	**1.841**	**0.344**	** < .0001**				**1.029**	**0.343**	**0.0027**
High	**4.428**	**0.356**	** < .0001**				**2.368**	**0.364**	** < .0001**
* Missing*	* − 0.005*	*0.019*	*0.8109*				*0.000*	*0.019*	*0.9816*
Income (individual) (thousand SEK)^d^									
Second quintile				**1.048**	**0.426**	**0.0139**	**0.899**	**0.427**	**0.0353**
Third quintile				**4.901**	**0.410**	** < .0001**	**4.495**	**0.414**	** < .0001**
Fourth quintile				**7.392**	**0.421**	** < .0001**	**6.844**	**0.427**	** < .0001**
Fifth quintile				**8.833**	**0.425**	** < .0001**	**8.041**	**0.440**	** < .0001**
* Missing*				***0.071***	***0.024***	***0.0032***	***0.071***	***0.024***	***0.003***
Adjusted R^2^	0.0762			0.0931			0.0948		
RMSE	17.94			17.78			17.76		
N	23,899			23,899			23,899		

Statistically significant estimates are shown in bold (< 0.05)

*RSE* Robust standard error

Reference groups: ^a^Men

^b^30‒34 years

^c^Low educational level

^d^First quintile (lowest)

^e^Educational levels: low= elementary school 9‒10 years; medium= secondary school 3‒4 years; high= more than 3‒4 years secondary school

The bell-shaped age pattern, with a peak in mean EQ VAS score in the age groups 65‒69 and 70‒74 years, was seen when adjusting for both education and income, with statistically significant differences for all age groups, except the age groups 60‒64 and 35‒39 years (Model 3). The pattern was similar in the other models, but when adjusting only for education (Model 1) the estimates for the ages up to 64 years and those 70‒74 years were not significant, and when adjusting only for income (Model 2), the estimates for the ages 60‒74 years were not significant.

Those unemployed and those with sick leave had a significantly lower mean EQ VAS score than those employed (Online Resource Table S13, Model 4).

#### Association between health-related behaviours, self-reported diseases, conditions and EQ VAS score

The association between health-related behaviours and self-reported diseases and mean EQ VAS score, adjusted for sex and age, for those aged 30‒104 years is shown in the Online Resource Table S14. Daily smoking, being physically active less than 150 min per week, or sitting more than 10 h per day significantly reduced the mean EQ VAS score (Model 1). When adjusting for education and income, the coefficient for risk consumption of alcohol was also significant; negatively associated with the EQ VAS score (Model 2). Increased number of diagnosed diseases reduced the mean EQ VAS score significantly (Model 3), and when adjusting for education and income, the associations remained, but with reduced coefficients (Model 4). The greatest coefficients for diseases diagnosed by a physician was seen for depression, followed by chronic obstructive pulmonary disease (COPD), both outpacing diabetes, hypertension and asthma by a factor of at least more than 2, with all coefficients being statistically significant (Model 5). The associations remained, with somewhat reduced estimates, after adjustments for education and income (Model 6).

Adjusting for health-related behaviours (Model 2), reduced the coefficients observed for education and income in Table 5 Model 3; the medium educational level and the second income quintile were no longer statistically significant. The statistically significant gradient in education and income remained after adjusting for number of diseases (Model 4) and when adjusting for the specific diseases (Model 6).

Being underweight, overweight or obese significantly reduced the mean EQ VAS score (Online Resource Table S15). The reduction on mean EQ VAS score from self-reported conditions, adjusted for sex, age, education and income, is shown in Online Resource Fig. S1. Having minor or severe self-reported conditions, with the exception of headache or migraine, significantly reduced the mean EQ VAS score; where the greatest coefficients were seen for severe tiredness, dejection, and aches or pain in back or in hip.

## Discussion

This study describes how HRQoL based on the EQ-5D-5L varies across subgroups and individual characteristics in the population, and investigates socioeconomic inequalities and individual heterogeneity in HRQoL, using data from 25,867 respondents aged 30‒104 [31], to the best of our knowledge one of the largest population-based surveys using EQ-5D-5L.

Generally, women reported worse health status than men, and health deteriorated with age, although the age gradient was bell-shaped, similar to a previous EQ-5D-3L population study in Sweden [12]. The present study shows evidence of the well-known socioeconomic gradient in health status [35]; more problems reported and lower mean observed EQ VAS scores, TTO and VAS index values among those with lower educational level, in lower income quintiles and those unemployed. These results are in line with findings of other studies investigating these relationships using EQ-5D-5L [e.g., 16, 26, 28]. Adjusting for health-related behaviours, diseases and self-reported conditions slightly reduced the differences between educational groups and income groups, but the socioeconomic differences largely remained.

Having a disease or a health condition was negatively associated with HRQoL, with the strongest association between EQ VAS score and those with depression diagnosed by a physician, or reporting conditions related to mental health and stress. These findings were also seen among adolescents in Sweden in Åström et al. [13] and in other studies [12, 36]. The mean EQ VAS score decreased with increasing number of diseases, findings similar to other studies see [e.g., 36, 37]. Daily smoking and being less physically active reduced HRQoL, as did deviations from normal BMI. Most problems were reported in the pain/discomfort dimension, similarly to findings in the Swedish population reference EQ-5D-3L [12] and EQ-5D-Y-3L [13] studies and other EQ-5D-5L reference studies summarised by Prevolnik Rupel et al. [19]. However, in the reference study for adolescents, girls reported most problems in the mood dimension [13].

In the present study, the proportion of the respondents reporting no problem on all dimensions (state 11111) was 24.1, which was much lower than in most other studies. For instance, in Trinidad and Tobago the proportion was 72% [29], in German studies 62% [22] and 31% [23], Bulgaria 50% [17], South Australia 43% [26], and in Slovenia 28% [19]. A lower proportion, 21%, reporting no problems was found in Quebec, Canada [16]. Among other factors influencing reporting problems, the age ranges differ across studies that makes it difficult to directly compare the proportions. As noted, our sample includes participants up to 104 years and health problems increase steeply among the oldest. Comparing to Swedish population studies, in the EQ-5D-3L study the proportion was 46% [12] and in the EQ-5D-Y-3L reference data for adolescents aged 13‒18 years the proportion was 45% [13].

The population reference data for the EQ-5D-5L in Sweden reported in the present study enable comparisons of results from other studies using EQ-5D-5L, and they also provide valuable inputs into health economic evaluations based on decision-analytic modelling. The HRQoL of the general population, by sex and age, is required in any model containing a disease-free, or “well”, health state. Furthermore, reductions in HRQoL associated with clinical events or health states, are often modelled as decrements from a population reference value.

As a future use, results derived by this study may also serve as socioeconomically and medically stratified population references for individual monitoring of HRQoL. Approaches using the EQ-5D instrument are under development for patient groups in oncology, asthma and diabetes [38–40]. With monitoring extending to other patient and population groups, population reference values are set to increase in relevance.

Another aspect, methods to value a health state may influence the burden of disease indicated for a population subgroup, as the following example shows. Compared to the overall population mean (Online Resource Table S3), people with asthma experience a loss of − 0.035 using the TTO index value but a loss of − 5.9 using the EQ VAS score; people with depression experience a loss of − 0.148 using the TTO index value but a loss of − 19.0 using the EQ VAS score (Table S4). With the EQ VAS score, the loss by depression exceeds that by asthma by a factor of 3.2, while with the TTO index value, this factor is 4.2, indicating a much higher relative burden. The valuation method may thus impact the relative burden for subgroups, and therefore the choice of method may impact decision making.

In this study, we identify important variation in problem levels reported as well as in overall valuation both related to socioeconomic inequality and to individual heterogeneity in health and health behaviour. The two components are also relevant from a policy perspective: systematic variation in HRQoL due to inequality requires responses directed at the socioeconomically disadvantaged groups, heterogeneity in the medical and behavioral factors requires search for more effective health promotion.

The equity implications of the findings of this study may be considerable and is an area to explore further. Adding a quality component to mortality differences across different subgroups in the population may reveal dramatic differences in life-time health prospects (see e.g., [41–44]). As an example, comparing highest income quintile vs. lowest shows a (crude) difference in 0.07‒0.10 depending on the method of measurement. A loss of 0.10 in HRQoL for 10 years may be translated to one year in full health lost. If lower income is also associated with increased mortality, the expected health loss over a lifetime may be substantial. For any health care system, taking health inequalities seriously such analyses will provide important input to health policy.

This study is larger and more comprehensive than studies only reporting standard population reference data, such as results by sex and broader age groups. Our study allows stratified analyses by 5-year age groups by sex, and analyses by socioeconomic characteristics, BMI and SRH, stratified by sex. Linking individual survey data to register data to obtain sociodemographic characteristics for each respondent is a strength. It was also possible to present results over the five severity levels, not being restricted to dichotomise into no problems vs any problems, by collapsing slight, moderate, severe and extreme problems into any problems. A shortcoming is that the descriptive analyses on education, income and economic activity were not stratified by age and that no sensitivity analyses with different dichotomisation of the severity levels were done. The fact that the sociodemographic composition of the CDUST Region is similar to the composition of the overall Swedish population implies that our results can be generalised to Sweden as a whole with respect to the studied age groups [32]. Due to the comprehensive data set, this study takes the advantage of the five levels in the EQ-5D-5L, which was developed to reduce the ceiling effect that has been found with the EQ-5D-3L [4]. To present HRQoL among the oldest old (95‒104 years) is another strength. However, as the survey did not include the EQ-5D-5L to those below 30 years, the lack of results for the youngest is a shortcoming of this study. A possible limitation could be the internal non-response rate in some groups; but due to the weak relationship between the specified variables and the response status, the implications are likely to be minor. The present study is based on the same data source as that used for the Swedish TTO and VAS EQ-5D-5L value sets [32], which is a strength as no other population is introduced. The individual respondents experiencing the health state report and value the health states. The cross-sectional design limits studying causality. Variables have been attributed to indicate either inequity or heterogeneity. Yet, other definitions may be used in specific research or policy contexts, e.g., when focusing on the biological aspect of age as one of heterogeneity, or when attributing multimorbidity to equity issues.

Findings of this study point out substantial socioeconomic inequalities and individual heterogeneity in HRQoL, which ought to be considered in decision making and in research. Thus, results add information that is especially important for equity considerations, an aspect often neglected in economic evaluation studies and in evidence-based decision making in health care [45].

## Conclusion

Based on a large population-based survey with 25,867 respondents aged 30‒104 years, EQ-5D-5L population reference data are now available for Sweden. This study investigates socioeconomic inequalities and individual heterogeneity in HRQoL, relating HRQoL to health-related behaviours, diseases and self-reported conditions. It presents respondents rating of their own overall health on the EQ VAS and results for different ways to assign a single value for each health state applying population-based value sets based on VAS valuation, and on TTO valuation allowing for calculation of QALYs. Yet, all measures refer to the individual’s own experienced health state. The results may be useful not only for comparisons with disease-specific populations and as inputs into health economic evaluation studies, but also for policy makers, concerned with both efficiency and equity aspects. The findings of inequality in HRQoL between socioeconomic groups indicate that the latter may be of importance in determining, and in implementing health policy targets.

## Supplementary Information

Below is the link to the electronic supplementary material.

Supplementary file1 (PDF 319 kb)

## Data Availability

Data sharing is not possible according to Swedish law.
