# Supplementary material for: Inequality and heterogeneity in health-related quality of life: findings based on a large sample of cross-sectional EQ-5D-5L data from the Swedish general population
Source: Qual Life Res. 2021 Oct 10;31(3):697–712. doi: 10.1007/s11136-021-02982-3 (PMC8921093; doi:10.1007/s11136-021-02982-3)
Supplement: Supplementary file 1 — Supplementary file1 (PDF 319 kb) [file 11136_2021_2982_MOESM1_ESM.pdf]

Electronic Supplementary Material (ESM)

ESM1.

Article title: Inequality and heterogeneity in health-related quality of life: findings based on a large sample of cross-sectional EQ-5D-5L data from the Swedish general population

Journal name: Quality of Life Research, 2021
doi: 10.1007/s11136-021-02982-3

Authors: Fitsum Sebsibe Teni, Ulf-G Gerdtham, Reiner Leidl, Martin Henriksson, Mimmi Åström, Sun Sun, Kristina Burström

Corresponding author:

Kristina Burström, PhD, Associate Professor

Health Outcomes and Economic Evaluation Research Group
Stockholm Centre for Healthcare Ethics
Department of Learning, Informatics, Management and Ethics
Karolinska Institutet
Tomtebodavägen 18a, SE-171 77 Stockholm, Sweden

email: kristina.burstrom@ki.se

ORCID: 0000-0001-9996-4317

CONTENT

Response rate, internal response rates, and information on non-respondents 3

Categorisation of demographic and socioeconomic information, and variables for health-related behaviours, diseases diagnosed by a physician and self-reported conditions.....3

Table S1 Sociodemographic characteristics of the populations in Sweden and the CDUST Regions, census data 2017 5

Table S2 Characteristics of respondents in Life & Health 2017 (n=25,867)............................6

Table S3 Proportion of health states reported by ten or more respondents (n=174) and the health state ‘55555’ 8

Table S4 Problems in EQ-5D-5L dimensions (%), mean and median EQ VAS score, mean TTO index value, and mean VAS index value, total sample, by age group (n=25,867)11

Table S5 Problems in EQ-5D-5L dimensions (%), mean and median EQ VAS score, mean TTO index value, and mean VAS index value, by sex, 30–64 years, by economic activity (n=11,303) 12

Table S6 Problems in EQ-5D-5L dimensions (%), mean and median EQ VAS score, mean TTO index value, and mean VAS index value, total sample, by diseases diagnosed by a physician and by self-reported conditions, 30–104 years 13

Table S7 Problems in EQ-5D-5L dimensions (%), mean and median EQ VAS score, mean TTO index value, and mean VAS index value, by sex, by Body Mass Index (BMI) groups, 30–104 years (n=24,694) 14

Table S8 Problems in EQ-5D-5L dimensions (%), mean and median EQ VAS score, mean TTO index value, and mean VAS index value, total sample, by self-reported stress and self-reported sickness (30–69 years), and number of diagnosed diseases (30–104 years)15

Table S9 Proportion of self-rated health (SRH) levels by sex, by age group (n=25,867).......16

Table S10 Problems in EQ-5D-5L dimensions (%), mean and median EQ VAS score, mean TTO index value, and mean VAS index value, total sample, by self-rated health (SRH) level, 30–104 years (n=25,677)..17

Table S11 Odds Ratios (OR) (multivariable logistic regression) for reporting any problems on the EQ-5D-5L dimensions and respondent’s sex, age, educational level and income, 30–104 years (n=25,867) 18

Table S12 Odds Ratios (OR) (multivariable logistic regression) for reporting any problems on the EQ-5D-5L dimensions and respondent’s sex, age, educational level, income and economic activity, 30–64 years (n=11,409)..19

Table S13 Ordinary Least Square (OLS) regression on mean EQ VAS score adjusted for sex, age, educational level, income and economic activity, 30–64 years (n=10,800) 20

Table S14 Ordinary Least Square (OLS) regression on mean EQ VAS score by health-related behaviours and diseases diagnosed by a physician, adjusted for sex, age, educational level and income, 30–104 years (n=23,899)...21

Table S15 Ordinary Least Square (OLS) regression on mean EQ VAS score by Body Mass Index (BMI) groups, adjusted for sex, age, educational level and income, 30–104 years (n=23,899)22

Figure S1 Ordinary Least Square (OLS) regression on mean EQ VAS score by self-reported conditions, adjusted for sex, age, educational level and income, estimates showing reduction in mean EQ VAS score with confidence intervals, 30–104 years (n=23,899) 23

SUPPLEMENTARY MATERIAL

Response rate, internal response rates, and information on non-respondents

The survey was sent out to 56,866 individuals aged 30-104 years in the regions/county councils Uppsala, Sörmland, Västmanland, Värmland, and Örebro in mid-Sweden (CDUST Region) [Burström et al., 2020, CDUST Region, 2018]. Potential participants were informed about the study and left with a choice either to participate or to refrain from participation. A returned survey was taken to rest on an informed consent to participate in the survey. The survey was answered by 26,789 individuals, yielding a 47.1% response rate. The response rate varied by age group: 55% (85 years and above); 70% (70-84 years); 53% (50-69 years; 34% (30-49 years) [CDUST Region, 2018]. Women had higher response rate than men, except among those 70 years and above where men had a slight higher response rate. Those born in Sweden had higher response rate than those born outside Sweden and those with higher education had higher response rate than those with lower educational level.

Missing responses on EQ-5D-5L dimensions were 1.4% for mobility, self-care and usual activities, respectively, 1.6% for pain/discomfort, 2.0% for anxiety/depression, and 8.7% for EQ VAS. After exclusion of individuals with missing responses in at least one dimension 3.4% (n=922) the present study used data from 25,867 individuals. Generally, internal non-respondents were older, had worse health status, lower educational level and lower income, and the proportion of women was higher compared to the respondents, however the effect size shows mainly weak level of strength of association between the specified variables and the response status [Burström et al., 2020].

Categorisation of demographic and socioeconomic information, and variables for health-related behaviours, diseases diagnosed by a physician and self-reported conditions

Demographic information was based on sex (men as reference group) and age categorised into 5-year age groups from 30–34 years as reference group until 95–104 years. Socioeconomic characteristics were based on: educational level categorised into low (elementary school 9–10 years) as reference group, medium (secondary school 3–4 years), and high (more than 3–4 years secondary school); individualised annual income divided into five groups of equal size with first quintile (lowest) group as reference group; economic activity only for the ages up to 64 years categorised into employed as reference group, studying/parental leave, unemployed, retired, and sick leave [for details see Burström et al., 2020].

Health-related behaviours included daily smoking (reference group: no or occasional smoking), risk consumption of alcohol (reference group: no risk consumption) [Babor et al., 2001], physical activity less than 150 minutes per week (reference group:150 minutes or more per week,), and number of hours sitting per day dichotomized into sitting 10 hours or more, and sitting less than 10 hours per day (reference group).

Self-reported information on Body Mass Index (BMI) was categorised into underweight (<18.5), normal (reference group: 18.5–24.9), overweight (25.0–29.9), and obesity class I (30.0–34.9), class II (35.0–39.9) or class III (40+) [WHO, 2000]. Respondents answered whether they had specific diseases diagnosed by a physician (reference group: individuals answering not having the specific disease) and whether they had minor or severe conditions (reference group: individuals answering that they did not have the specific condition). Global Self-Rated Health (SRH) was assessed from answers to the question: ‘In your opinion, how is your health status? Is it very good, good, neither good nor bad, bad or very bad?’ Those answering neither good nor bad, bad or very bad were categorised as having ‘less than good SRH’.

Babor, T., Higgins-Biddle, J. C., Aaunders, J. B., & Monteiro, M. G. (2001). Audit. The alcohol disorders identification test. Guidelines for use in primary care. (2nd ed.). World Health Organization.

Burström, K., Teni, F. S., Gerdtham, U, G., Leidl, R., Helgesson, G., Rolfson, O., & Henriksson, M. (2020). Experience-based Swedish TTO and VAS value sets for EQ-5D-5L health states. *PharmacoEconomics*, *38*(8), 839–856.

CDUST Region 2018. Liv & hälsa 2017 i Mellansverige: resultat från en undersökning om livsvillkor, levnadsvanor och hälsa (in Swedish). [Life and health 2017: results from a survey on living conditions, health-related behaviours and health]. Available from: https://www.regionvarmland.se/globalassets/global/om-regionen/pressrum/pressmeddelanden/2018/februari/rv-liv-och-halsa-2017.pdf (accessed 8 June 2021).

WHO consultation on obesity. Obesity: Preventing and managing the global epidemic, Report of a WHO Consultation. Geneva, Switzerland, WHO 2000. http://whqlibdoc.who.int/trs/WHO_TRS_894.pdf. (accessed 15 Jan 2021).

Table S1 Sociodemographic characteristics of the populations in Sweden and the CDUST Regions, census data 2017

Variable	Sweden		Uppsala		Södermanland		Västmanland		Värmland		Örebro	
	**Men**	**Women**	**Men**	**Women**	**Men**	**Women**	**Men**	**Women**	**Men**	**Women**	**Men**	**Women**
**Proportion of men and women (%)**	50.2	49.8	50.0	50.0	50.1	49.9	50.3	49.7	50.3	49.7	50.1	49.9
**Average age** (years)	41.2		40.1		42.3		49.7		43.5		41.6	
**Educational level** [a]												
Low	21.2	19.0	19.3	16.5	24.6	23.2	22.8	21.6	22.3	21.5	23.2	21.0
Medium	44.8	40.2	41.9	37.1	49.8	43.6	48.8	43.0	50.8	47.1	49.2	42.7
High	31.1	38.6	36.1	44.4	23.4	31.2	26.0	33.2	24.2	35.1	25.6	34.4
No information	2.9	2.1	2.7	1.9	2.3	1.9	2.3	1.9	2.7	2.1	2.1	1.8
**Income** (median equivalised) (SEK) [b]	256,600		260,700		244,100		250,200		235,500		240,800	

[a] low= elementary school 9–10 years; medium= secondary school 3–4 years; high= more than 3–4 years secondary school

[b] 1 SEK = 9.63 Euro (2017 prices) (Swedish Riksbank)

Swedish Riksbank. Annual average exchange rates, December, 2017. Accessed 1 August, 2021. https://www.riksbank.se/en-gb/statistics/search-interest--exchange-rates/annual-average-exchange-rates/?y=2017&m=12&s=Dot&f=y

The table is published in Online Resource ESM_1 to the article Burström, K., Teni, F. S., Gerdtham, U, G., Leidl, R., Helgesson, G., Rolfson, O., & Henriksson, M. (2020). Experience-based Swedish TTO and VAS value sets for EQ-5D-5L health states. *PharmacoEconomics, 38*(8), 839–856

Table S2 Characteristics of respondents in Life & Health 2017 (n=25,867)

Variable	%	n
**Sex**		
Men	47.4	12,249
Women	52.6	13,618
**Mean age (years) [SD]**		64.3 [16.0]
**Age group (years)**		
30-34	4.4	1,127
35-39	4.6	1,202
40-44	5.7	1,473
45-49	6.7	1,722
50-54	7.0	1,824
55-59	7.4	1,917
60-64	8.3	2,144
65-69	10.2	2,647
70-74	17.4	4,489
75-79	11.5	2,972
80-84	6.7	1,723
85-89	6.8	1,762
90-94	2.7	703
95-104	0.6	162
**Educational level** [a]		
Low	23.2	6,005
Medium	42.6	11,027
High	33.6	8,694
Missing	0.6	141
**Income**		
First quintile (lowest)	19.9	5,159
Second quintile	20.0	5,160
Third quintile	19.9	5,159
Fourth quintile	20.0	5,160
Fifth quintile (highest)	19.9	5,159
Missing	0.3	70
**Economic activity (30–64 years)**		
Employed	83.7	9,549
Studying/parental leave	3.5	403
Unemployed	3.5	394
Retired	1.1	122
Sick leave	7.3	835
Missing	0.9	106
**Self-rated health (SRH)**		
Very good	15.7	4,064
Good	48.9	12,650
Neither good or bad	28.5	7,378
Bad	5.1	1,323
Very bad	1.0	262
Missing	0.7	190
**Less than good SRH**	34.6	9,153
**Smoking daily**	8.2	2,110
**Risk consumption of alcohol**	8.8	2,286
**Active less than 150 minutes per week**	39.9	10,118
**Sitting (hours per day)**		
More than 12	3.2	829
10 to 12	8.3	2,153
7 to 9	22.6	5,851
4 to 6	43.2	11,169
1 to 3	18.6	4,821
Less than 1	0.9	220
Sit/lie for more than 12 hours due to disability	1.7	434
Missing	1.5	390
**BMI (self-reported height and weight)**		
Underweight	1.5	383
Normal	37.4	9,685
Overweight	37.8	9,785
Obese class I	13.6	3,519
Obese class II	3.4	886
Obese class III	1.7	436
Missing	4.5	1,173

**Diseases diagnosed by a physician**		
Asthma	7.0	1,803
COPD	3.4	870
Depression	7.3	1,891
Diabetes	10.0	2,575
Hypertension	32.2	8,321
**Number of diseases**		
One	31.6	8,182
Two	9.1	2,349
Three	1.5	383
Four	0.2	63
Five	0.1	16
Missing	5.3	1,367
**Self-reported conditions**		
**Headache or migraine**		
Minor	15.8	4,077
Severe	2.2	572
**Ache/pain in shoulders/neck**		
Minor	41.2	10,644
Severe	6.5	1,668
**Ache/pain in elbows/legs/knees**		
Minor	41.3	10,693
Severe	7.7	1,999
**Ache/pain in back/hip or sciatica**		
Minor	41.1	10,631
Severe	8.9	2,306
**Recurrent stomach/bowel problems**		
Minor	21.4	5,523
Severe	3.7	950
**Eczema**		
Minor	11.9	3,069
Severe	1.1	277
**Dejection**		
Minor	22.1	5,723
Severe	2.5	649
**Anxiety/worry**		
Minor	23.4	6,049
Severe	2.9	742
**Sleeping difficulties**		
Minor	28.7	7,432
Severe	5.9	1,512
**Tiredness**		
Minor	39.9	10,310
Severe	7.0	1,811
**Stress (30–69 years)**		
Not at all	43.7	6,142
To some extent	43.9	6,170
Quite a lot	9.1	1,285
Very much	2.8	391
Missing	0.5	68
**Sick for more than 29 continuous days in the past 12 months (30–69 years)**		
No, I have not been sick for a continuous period of 29 days or more	65.1	9,151
Yes, for problems with my back, neck, joints or muscles	2.3	317
Yes, for exhaustion, fatigue, depression, stress	3.2	455
Yes, for other mental health problems (including insomnia)	0.9	120
Yes, for cardiovascular disease, stroke	0.4	60
Yes, for accidents, injury	1.3	187
Yes, for other reasons	2.5	344
Missing (across all variables)	21.3	3,691
**EQ VAS score (mean) [SD]**	76.1 [18.7]	
**EQ VAS score (median) [IQR]**	80.0 [20.0]	
**TTO index value (mean) [SD]**	0.892 [0.114]	
**VAS index value (mean) [SD]**	75.9 [14.0]	

[a] low= elementary school 9–10 years; medium= secondary school 3–4 years; high= more than 3–4 years secondary school

The table is adapted from Table 2 in Burström, K., Teni, F. S., Gerdtham, U, G., Leidl, R., Helgesson, G., Rolfson, O., & Henriksson, M. (2020). Experience-based Swedish TTO and VAS value sets for EQ-5D-5L health states. *PharmacoEconomics, 38*(8), 839–856.

Table S3 Proportion of health states reported by ten or more respondents (n=174) and the health state '55555'

Health state	n	%	Cumulative %
**11111**	6234	24.1	24.1
**11121**	4445	17.2	41.3
**11122**	1987	7.7	49.0
**11112**	1213	4.7	53.7
**11131**	778	3.0	56.7
**21121**	751	2.9	59.6
**21221**	461	1.8	61.3
**11132**	441	1.7	63.1
**21231**	409	1.6	64.6
**11221**	334	1.3	65.9
**21232**	353	1.4	67.3
**21122**	300	1.2	68.5
**11222**	296	1.1	69.6
**21131**	290	1.1	70.7
**21222**	288	1.1	71.8
**31231**	212	0.8	72.6
**11123**	194	0.7	73.4
**11232**	186	0.7	74.1
**31232**	172	0.7	74.8
**21132**	168	0.6	75.4
**11231**	153	0.6	76.0
**31331**	141	0.5	76.6
**21111**	133	0.5	77.1
**31332**	129	0.5	77.6
**11113**	118	0,5	78.0
**31131**	113	0.4	78.5
**11133**	108	0.4	78.9
**31221**	94	0.4	79.3
**32332**	84	0.3	79.6
**22232**	77	0.3	79.9
**31121**	70	0.3	80.1
**32232**	70	0.3	80.4
**21233**	69	0.3	80.7
**11223**	68	0.3	80.9
**21211**	66	0.3	81.2
**22222**	64	0.2	81.5
**22221**	62	0.2	81.7
**31132**	62	0.2	81.9
**31222**	57	0.2	82.2
**22231**	56	0.2	82.4
**11233**	55	0.2	82.6
**11211**	54	0.2	82.8
**11212**	54	0.2	83.0
**32231**	53	0.2	83.2
**21331**	50	0.2	83.4
**32331**	49	0.2	83.6
**21332**	47	0.2	83.8
**33332**	41	0.2	83.9
**21223**	39	0.2	84.1
**31321**	36	0.1	84.2
**11332**	35	0.1	84.4
**31342**	35	0.1	84.5
**33331**	35	0.1	84.6
**21112**	32	0.1	84.7
**41231**	32	0.1	84.9
**31211**	31	0.1	85.0
**31233**	31	0.1	85.1
**11124**	30	0.1	85.2
**11141**	30	0.1	85.3
**21242**	30	0.1	85.5
**12221**	29	0.1	85.6

**55532**	29	0.1	85.7
**11331**	28	0.1	85.8
**21212**	28	0.1	85.9
**32222**	28	0.1	86.0
**43442**	28	0.1	86.1
**31122**	26	0.1	86.2
**31242**	26	0.1	86.3
**31333**	26	0.1	86.4
**43332**	26	0.1	86.5
**44443**	26	0.1	86.6
**21123**	25	0.1	86.7
**22332**	25	0.1	86.8
**31241**	25	0.1	86.9
**32342**	25	0.1	87.0
**33333**	24	0.1	87.1
**42331**	24	0.1	87.2
**11322**	23	0.1	87.3
**42442**	23	0.1	87.4
**44442**	23	0.1	87.5
**21133**	22	0.1	87.5
**21321**	22	0.1	87.6
**21322**	22	0.1	87.7
**31341**	22	0.1	87.8
**12121**	21	0.1	87.9
**12231**	21	0.1	88.0
**31322**	21	0.1	88.0
**32233**	21	0.1	88.1
**41332**	21	0.1	88.2
**11333**	20	0.1	88.3
**21241**	20	0.1	88.4
**32333**	20	0.1	88.4
**41331**	20	0.1	88.5
**43432**	20	0.1	88.6
**43443**	20	0.1	88.7
**11321**	19	0.1	88.7
**41232**	19	0.1	88.8
**43331**	19	0.1	88.9
**11234**	18	0.1	89.0
**21333**	18	0.1	89.0
**31111**	18	0.1	89.1
**31431**	18	0.1	89.2
**41441**	18	0.1	89.2
**43342**	18	0.1	89.3
**11114**	17	0.1	89.4
**11134**	17	0.1	89.4
**11242**	17	0.1	89.5
**11323**	17	0.1	89.6
**21234**	17	0.1	89.6
**22331**	17	0.1	89.7
**22333**	17	0.1	89.8
**32221**	17	0.1	89.8
**42432**	17	0.1	89.9
**11142**	16	0.1	90.0
**12232**	16	0.1	90.0
**22233**	16	0.1	90.1
**41241**	16	0.1	90.1
**41342**	16	0.1	90.2
**55522**	16	0.1	90.3
**11213**	15	0.1	90.3
**21142**	15	0.1	90.4
**21243**	15	0.1	90.4
**32241**	15	0.1	90.5
**32322**	15	0.1	90.6
**33443**	15	0.1	90.6
**41341**	15	0.1	90.7

**11313**	14	0.1	90.7
**21141**	14	0.1	90.8
**21342**	14	0.1	90.8
**22322**	14	0.1	90.9
**31243**	14	0.1	90.9
**31343**	14	0.1	91.0
**41432**	14	0.1	91.1
**42332**	14	0.1	91.1
**42342**	14	0.1	91.2
**43444**	14	0.1	91.2
**44444**	14	0.1	91.3
**55533**	14	0.1	91.3
**11241**	13	0.1	91.4
**32242**	13	0.1	91.4
**32321**	13	0.1	91.5
**32341**	13	0.1	91.5
**32432**	13	0.1	91.6
**33342**	13	0.1	91.6
**42443**	13	0.1	91.7
**43433**	13	0.1	91.7
**44432**	13	0.1	91.8
**55531**	13	0.1	91.8
**11334**	12	0.0	91.9
**22223**	12	0.0	91.9
**31311**	12	0.0	92.0
**31442**	12	0.0	92.0
**33431**	12	0.0	92.1
**41242**	12	0.0	92.1
**41443**	12	0.0	92.1
**43431**	12	0.0	92.2
**12111**	11	0.0	92.2
**21341**	11	0.0	92.3
**21432**	11	0.0	92.3
**22211**	11	0.0	92.4
**31432**	11	0.0	92.4
**32442**	11	0.0	92.4
**33322**	11	0.0	92.5
**43333**	11	0.0	92.5
**11143**	10	0.0	92.6
**11343**	10	0.0	92.6
**22242**	10	0.0	92.7
**32433**	10	0.0	92.7
**32443**	10	0.0	92.7
**33432**	10	0.0	92.8
**41132**	10	0.0	92.8
**41442**	10	0.0	92.8
**44433**	10	0.0	92.9
**55542**	10	0.0	92.9
**…**	…	…	…
**55555**	6	0.0	–

In total 896 health states were reported in Life & Health 2017
174 health states were reported by ten or more respondents
291 health states were reported by five or more respondents

Table S4 Problems in EQ-5D-5L dimensions (%), mean and median EQ VAS score, mean TTO index value, and mean VAS index value, total sample, by age group (n=25,867)

**EQ-5D-5L dimension**	**Men and women age group (years)**													
	**Total**	**30-34**	**35-39**	**40-44**	**45-49**	**50-54**	**55-59**	**60-64**	**65-69**	**70-74**	**75-79**	**80-84**	**85-89**	**90-94**	**95-104**
	n=25,867	n=1,127	n=1,202	n=1,473	n=1,722	n=1,824	n=1,917	n=2,144	n=2,647	n=4,489	n=2,972	n=1,723	n=1,762	n=703	n=162
**Mobility**															
No problems	67.6	89.1	89.7	87.7	84.5	80.1	76.3	73.0	72.0	67.5	58.0	45.7	31.5	21.8	11.7
Slight problems	17.2	8.0	8.0	8.6	11.2	12.1	14.1	17.2	17.4	18.5	22.3	25.2	28.0	24.9	19.1
Moderate problems	9.7	2.2	1.7	2.6	3.1	5.6	6.6	6.9	6.8	9.7	13.2	18.6	23.6	29.6	21.6
Severe problems	4.4	0.5	0.6	1.0	1.0	2.0	2.8	2.5	3.2	3.4	5.7	8.4	12.9	16.8	26.5
Extreme problems	1.2	0.2	0.1	0.1	0.2	0.2	0.2	0.4	0.6	1.0	0.9	2.2	4.0	7.0	21.0
**Self-care**															
No problems	89.2	96.4	98.3	96.2	95.6	93.4	92.1	92.7	93.1	91.1	88.0	81.0	72.4	55.5	34.0
Slight problems	6.0	2.9	1.3	2.6	3.0	4.8	5.7	4.8	4.5	5.8	7.3	10.4	11.3	16.1	15.4
Moderate problems	2.8	0.7	0.2	0.7	1.0	1.5	1.7	1.6	1.6	1.9	3.0	4.9	9.1	15.2	14.8
Severe problems	1.1	0.0	0.0	0.4	0.2	0.2	0.4	0.6	0.7	0.6	1.1	2.2	4.2	6.5	16.0
Extreme problems	0.8	0.0	0.1	0.1	0.1	0.0	0.1	0.3	0.1	0.6	0.6	1.6	3.1	6.7	19.8
**Usual activities**															
No problems	69.2	81.1	83.0	81.2	78.9	76.9	73.6	72.3	75.3	73.4	64.4	52.8	41.3	28.6	19.1
Slight problems	18.2	13.3	11.2	12.1	14.5	13.9	17.0	18.9	17.2	17.1	21.9	26.0	27.6	24.9	22.2
Moderate problems	7.1	4.0	3.7	4.2	3.9	5.2	5.1	5.2	4.4	6.1	8.5	12.9	16.2	20.5	13.0
Severe problems	3.6	2.0	1.8	2.0	2.2	3.1	3.4	3.0	2.5	2.1	3.6	4.8	8.7	13.7	16.7
Extreme problems	2.0	0.6	0.3	0.5	0.6	1.0	0.9	0.6	0.6	1.3	1.6	3.5	6.2	12.4	29.0
**Pain/discomfort**															
No problems	32.0	49.2	49.2	42.4	40.3	34.6	31.4	30.0	30.1	32.1	26.8	22.7	20.0	16.5	15.4
Slight problems	39.8	36.6	37.5	40.7	37.8	40.7	41.1	42.1	43.0	40.2	40.8	38.3	36.2	35.7	32.1
Moderate problems	22.5	10.8	10.7	13.2	17.1	18.1	20.8	22.7	22.7	22.5	26.6	31.2	34.3	37.3	39.5
Severe problems	5.2	3.1	2.3	3.5	4.1	5.9	6.1	4.9	4.0	4.7	5.3	7.2	8.6	9.8	11.1
Extreme problems	0.4	0.3	0.2	0.1	0.7	0.7	0.6	0.3	0.2	0.4	0.4	0.6	0.8	0.7	1.8
**Anxiety/depression**															
No problems	62.8	48.4	54.2	55.3	59.5	58.0	64.3	67.3	71.6	68.7	65.8	63.0	57.1	54.5	43.8
Slight problems	28.8	35.6	34.6	33.1	30.8	31.8	26.3	26.1	23.6	25.9	27.9	28.4	32.4	34.8	34.6
Moderate problems	6.0	10.5	7.9	8.5	6.6	7.0	6.5	4.4	3.7	4.0	5.2	6.0	7.8	7.4	16.0
Severe problems	2.0	4.2	2.6	2.4	2.5	2.8	2.4	1.8	0.9	1.3	0.8	2.3	2.2	3.3	4.9
Extreme problems	0.4	1.3	0.7	0.7	0.6	0.4	0.4	0.5	0.2	0.2	0.3	0.3	0.5	0.0	0.6
**EQ VAS score (mean) [SD]**	76.1 [18.7]	79.0 [16.0]	78.6 [16.0]	78.4 [16.7]	78.1 [17.1]	77.6 [17.8]	78.0 [18.2]	78.8 [17.1]	79.8 [16.9]	78.3 [17.8]	74.6 [18.5]	70.0 [20.0]	64.9 [21.2]	60.1 [22.6]	55.1 [24.1]
**EQ VAS score (median) [IQR]**	80.0 [20.0]	80.0 [19.0]	80.0 [20.0]	80.0 [20.0]	80.0 [20.0]	80.0 [20.0]	80.0 [20.0]	80.0 [20.0]	85.0 [15.0]	80.0 [20.0]	80.0 [25.0]	75.0 [25.0]	70.0 [30.0]	60.0 [30.0]	55.0 [40.0]
**TTO index value (mean) [SD]**	0.892 [0.114]	0.911 [0.099]	0.922 [0.086]	0.916 [0.093]	0.913 [0.100]	0.903 [0.109]	0.901 [0.111]	0.904 [0.103]	0.912 [0.094]	0.904 [0.103]	0.887 [0.110]	0.858 [0.130]	0.824 [0.143]	0.783 [0.149]	0.718 [0.164]
**VAS index value (mean) [SD]**	75.9 [14.0]	78.4 [12.3]	79.8 [11.0]	78.9 [11.7]	78.6 [12.2]	77.4 [13.2]	77.1 [13.4]	77.4 [12.7]	78.2 [12.0]	77.3 [13.0]	75.0 [13.7]	71.5 [15.6]	67.4 [16.8]	62.7 [17.2]	54.6 [18.9]

Table S5 Problems in EQ-5D-5L dimensions (%), mean and median EQ VAS score, mean TTO index value, and mean VAS index value, by sex, 30–64 years, by economic activity (n=11,303)

EQ-5D-5L dimension	Total sample (%)					Men (%)					Women (%)				
	Employed	Studying /parental leave	Unemployed	Retired	Sick leave	Employed	Studying /parental leave	Unemployed	Retired	Sick leave	Employed	Studying /parental leave	Unemployed	Retired	Sick leave
	n=9,549	n=403	n=394	n=122	n=835	n=4,252	n=107	n=183	n=59	n=257	n=5,297	n=296	n=211	n=63	n=578
**Mobility**															
No problems	85.3	86.6	61.7	72.1	48.7	86.5	85.1	65.6	76.3	48.6	84.3	87.2	58.3	68.3	48.8
Slight problems	10.8	7.9	20.6	18.0	22.3	10.0	8.4	16.4	17.0	19.1	11.5	7.8	24.2	19.1	23.7
Moderate problems	3.1	3.0	12.4	5.7	17.7	2.8	3.7	11.5	1.7	19.8	3.3	2.7	13.3	9.5	16.8
Severe problems	0.7	2.5	4.8	4.1	9.3	0.7	2.8	6.0	5.1	10.9	0.8	2.4	3.8	3.2	8.7
Extreme problems	0.1	0.0	0.5	0.0	1.9	0.1	0.0	0.6	0.0	1.6	0.1	0.0	0.5	0.0	2.1
**Self-care**															
No problems	96.9	97.5	84.5	95.1	71.7	96.7	95.3	85.8	93.2	69.3	97.1	98.3	83.4	96.8	72.8
Slight problems	2.6	1.5	10.7	2.5	16.7	2.8	2.8	8.7	5.1	18.3	2.5	1.0	12.3	0.0	15.9
Moderate problems	0.4	0.5	3.8	1.6	8.3	0.5	0.9	3.8	1.7	10.5	0.4	0.3	3.8	1.6	7.3
Severe problems	0.1	0.5	0.8	0.0	2.5	0.1	0.9	1.1	0.0	1.2	0.1	0.3	0.5	0.0	3.1
Extreme problems	0.0	0.0	0.3	0.8	0.8	0.0	0.0	0.6	0.0	0.8	0.0	0.0	0.0	1.6	0.9
**Usual activities**															
No problems	81.8	80.7	57.4	73.8	35.5	84.4	82.2	60.1	79.7	36.6	79.7	80.1	55.0	68.3	35.0
Slight problems	13.7	12.9	21.3	21.3	23.7	12.1	9.4	21.3	15.3	28.0	15.0	14.2	21.3	27.0	21.8
Moderate problems	3.2	4.7	11.4	3.3	17.7	2.3	6.5	10.4	5.1	14.0	3.8	4.1	12.3	1.6	19.4
Severe problems	1.1	1.7	8.1	1.6	17.6	0.9	1.9	6.0	0.0	15.2	1.3	1.7	10.0	3.2	18.7
Extreme problems	0.2	0.0	1.8	0.0	5.5	0.3	0.0	2.2	0.0	6.2	0.2	0.0	1.4	0.0	5.2
**Pain/discomfort**															
No problems	39.9	48.4	28.9	32.0	16.7	44.2	56.1	35.5	40.7	19.5	36.5	45.6	23.2	23.8	15.4
Slight problems	41.6	34.2	35.3	41.8	24.6	40.6	31.8	31.2	33.9	26.9	42.4	35.1	38.9	49.2	23.5
Moderate problems	15.6	13.4	22.8	24.6	32.9	13.0	9.4	20.8	23.7	32.3	17.7	14.9	24.6	25.4	33.2
Severe problems	2.7	3.5	10.2	1.6	22.6	2.0	2.8	9.8	1.7	18.3	3.3	3.7	10.4	1.6	24.6
Extreme problems	0.1	0.5	2.8	0.0	3.2	0.1	0.0	2.7	0.0	3.1	0.1	0.7	2.8	0.0	3.3
**Anxiety/depression**															
No problems	62.6	52.6	32.2	77.9	36.1	68.7	57.0	36.6	79.7	37.7	57.8	51.0	28.4	76.2	35.3
Slight problems	30.0	33.3	39.3	21.3	30.9	25.7	33.6	32.2	20.3	29.6	33.5	33.1	45.5	22.2	31.5
Moderate problems	5.7	9.4	15.7	0.8	17.8	4.3	6.5	16.9	0.0	21.4	6.8	10.5	14.7	1.6	16.3
Severe problems	1.5	4.2	8.1	0.0	11.7	1.2	2.8	8.2	0.0	9.0	1.7	4.7	8.1	0.0	13.0
Extreme problems	0.2	0.5	4.6	0.0	3.5	0.2	0.0	6.0	0.0	2.3	0.2	0.7	3.3	0.0	4.0
**EQ VAS score (mean) [SD]**	80.3 [14.8]	77.7 [17.6]	68.1 [23.1]	80.9 [13.4]	59.4 [24.3]	81.3 [14.0]	80.6 [15.3]	66.8 [25.0]	80.2 [15.1]	61.8 [24.4]	79.6 [15.5]	76.7 [18.3]	69.2 [21.4]	81.6 [11.7]	58.4 [24.2]
**EQ VAS score (median) [IQR]**	83.0 [15.0]	80.0 [20.0]	75.0 [35.0]	85.0 [20.0]	60.0 [40.0]	85.0 [15.0]	85.0 [15.0]	75.0 [38.5]	80.0 [20.0]	69.5 [40.0]	80.0 [17.0]	80.0 [20.0]	75.0 [35.0]	85.0 [20.0]	60.0 [40.0]
**TTO index value (mean) [SD]**	0.924 [0.077]	0.912 [0.095]	0.832 [0.159]	0.921 [0.077]	0.768 [0.171]	0.932 [0.073]	0.920 [0.090]	0.836 [0.165]	0.928 [0.075]	0.782 [0.169]	0.918 [0.080]	0.909 [0.097]	0.828 [0.154]	0.914 [0.079]	0.762 [0.172]
**VAS index value (mean) [SD]**	79.9 [10.2]	78.5 [12.0]	68.8 [18.0]	79.4 [10.5]	61.6 [18.7]	81.1 [9.6]	79.7 [11.4]	69.3 [18.9]	80.4 [10.4]	63.1 [18.6]	78.9 [10.5]	78.1 [12.3]	68.4 [17.3]	78.4 [10.6]	61.0 [18.7]

Table S6 Problems in EQ-5D-5L dimensions (%), mean and median EQ VAS score, mean TTO index value, and mean VAS index value, total sample, by diseases diagnosed by a physician and by self-reported conditions, 30–104 years

	**Diseases diagnosed by a physician**					**Self-reported conditions**									
**EQ-5D-5L dimension**	Asthma	COPD	Depression	Diabetes	Hyper-tension	Headache or migraine	Ache/pain in shoulders/ neck	Ache/pain in elbows/legs/ knees	Ache/pain in back/hip	Recurrent stomach /bowel problems	Eczema	Dejection	Anxiety/ worry	Tiredness	Sleeping difficulties
	n=1,803	n=870	n=1,891	n=2,575	n=8,321	n=4,649	n=12,312	n=12,692	n=12,937	n=6,473	n=3,346	n=6,372	n=6,791	n=12,121	n=8,944
**Mobility**															
No problems	58.0	33.9	47.0	46.8	53.2	65.1	61.4	52.7	55.8	53.4	62.2	53.1	56.4	54.7	56.8
Slight problems	20.5	27.1	20.2	23.8	23.1	18.7	20.1	24.6	22.9	22.7	19.6	20.7	19.8	21.7	21.6
Moderate problems	13.2	22.1	18.6	16.7	15.2	9.7	12.0	14.5	13.7	13.8	10.3	14.9	13.5	14.3	13.3
Severe problems	6.6	13.5	10.5	10.1	7.2	5.2	5.2	6.9	6.4	7.7	6.6	8.5	7.9	7.4	6.9
Extreme problems	1.7	3.5	3.8	2.6	1.4	1.3	1.3	1.5	1.3	2.4	1.4	2.9	2.5	1.9	1.4
**Self-care**															
No problems	85.2	71.8	70.9	78.2	84.3	86.3	85.8	84.1	85.0	80.6	85.5	78.2	80.2	82.1	84.0
Slight problems	8.4	14.9	13.9	12.1	8.8	7.4	8.1	8.9	8.6	10.1	7.6	10.8	9.9	9.6	8.8
Moderate problems	3.8	7.6	8.5	5.3	4.2	3.8	3.8	4.3	4.0	5.1	4.0	6.0	5.3	4.8	4.5
Severe problems	1.6	3.1	4.4	2.8	1.6	1.7	1.5	1.6	1.5	2.4	1.6	2.9	2.7	2.0	1.7
Extreme problems	1.0	2.5	2.3	1.6	1.0	0.8	0.9	1.0	1.0	1.8	1.2	2.1	2.0	1.5	1.0
**Usual activities**															
No problems	56.9	37.6	35.0	52.8	59.0	60.1	60.9	56.2	58.0	52.3	62.6	48.3	51.5	53.2	55.8
Slight problems	24.4	30.5	28.3	24.9	23.7	21.6	22.9	25.4	24.5	24.6	20.6	25.2	24.3	25.5	24.3
Moderate problems	10.2	16.4	17.0	11.7	9.8	9.5	9.4	10.5	9.8	12.0	8.8	13.3	12.0	11.6	11.1
Severe problems	6.1	10.6	13.6	6.8	5.0	6.2	4.7	5.4	5.4	7.0	5.5	8.6	7.9	6.4	6.3
Extreme problems	2.5	4.9	6.1	3.7	2.5	2.5	2.2	2.5	2.3	4.0	2.5	4.6	4.3	3.3	2.6
**Pain/discomfort**															
No problems	20.0	15.4	13.5	20.9	22.2	19.7	14.8	10.9	12.0	14.7	23.5	17.3	18.8	18.8	19.4
Slight problems	38.6	31.4	30.4	36.5	40.3	38.9	44.7	45.7	45.2	38.6	41.7	36.9	37.7	39.5	38.4
Moderate problems	30.6	39.8	37.5	32.0	29.5	29.5	31.7	34.0	33.2	34.4	25.9	33.1	31.8	31.7	31.4
Severe problems	9.2	11.2	15.9	9.7	7.5	10.7	8.1	8.7	8.8	11.3	8.1	11.3	10.4	9.1	9.8
Extreme problems	1.6	2.3	2.7	0.9	0.6	1.2	0.7	0.8	0.8	1.0	0.8	1.3	1.2	0.9	1.0
**Anxiety/depression**															
No problems	53.6	49.1	5.1	60.1	61.9	42.1	53.1	55.1	54.7	43.6	54.2	5.1	7.4	42.5	43.1
Slight problems	32.8	36.1	39.3	29.8	29.4	39.3	35.2	33.7	34.0	39.1	32.9	64.4	64.1	42.1	40.4
Moderate problems	9.2	10.2	33.8	7.0	6.3	12.2	8.0	7.9	7.9	11.8	9.1	21.5	21.0	10.8	11.2
Severe problems	3.7	3.5	17.3	2.7	2.0	5.1	3.1	2.9	2.9	4.5	3.3	7.5	7.0	3.8	4.3
Extreme problems	0.6	1.2	4.4	0.4	0.4	1.3	0.6	0.5	0.5	0.9	0.7	1.5	1.5	0.8	1.0
**EQ VAS score (mean) [SD]**	70.2 (20.2)	61.0 (21.7)	57.1 (22.1)	68.4 (20.0)	71.8 (19.4)	69.6 (20.8)	71.9 (19.3)	71.1 (19.6)	71.4 (19.4)	67.4 (20.8)	72.1 (20.1)	64.0 (20.9)	65.5 (21.0)	68.2 (19.9)	69.3 (19.8)
**EQ VAS score (median) [IQR]**	75.0 [25.0]	60.0 [30.0]	60.0 [35.0]	70.0 [30.0]	75.0 [25.0]	75.0 [25.0]	75.0 [25.0]	75.0 [25.0]	75.0 [25.0]	70.0 [33.5]	75.0 [30.0]	70.0 [30.0]	70.0 [30.0]	75.0 [25.0]	75.0 [25.0]
**TTO index value (mean) [SD]**	0.857 (0.136)	0.809 (0.151)	0.744 (0.169)	0.849 (0.137)	0.869 (0.125)	0.852 (0.141)	0.866 (0.126)	0.857 (0.128)	0.861 (0.127)	0.837 (0.142)	0.868 (0.134)	0.805 (0.147)	0.815 (0.146)	0.846 (0.134)	0.848 (0.136)
**VAS index value (mean) [SD]**	71.4 (15.9)	65.4 (17.3)	58.2 (17.6)	70.4 (16.2)	72.8 (15.0)	70.8 (16.2)	72.3 (14.7)	71.1 (14.9)	71.5 (14.8)	68.7 (16.3)	72.8 (15.7)	64.6 (16.1)	65.8 (16.1)	69.7 (15.6)	70.1 (15.6)

Comparing proportions of any problems (chi-square tests) and mean EQ VAS scores, TTO index values and VAS index values (independent t-tests) between groups with a specific disease or condition with groups without having the specific disease or condition showed statistically significant differences in all comparisons. Slight, moderate, severe and extreme problems were collapsed into any problems for the chi-square test.

Table S7 Problems in EQ-5D-5L dimensions (%), mean and median EQ VAS score, mean TTO index value, and mean VAS index value, by sex, by Body Mass Index (BMI) groups, 30–104 years (n=24,694)

**EQ-5D-5L dimension**	**Total sample (%)**						**Men (%)**						**Women (%)**					
	**BMI**						**BMI**						**BMI**					
	Underweight	Normal	Overweight	Obese class I	Obese class II	Obese class III	Underweight	Normal	Overweight	Obese class I	Obese class II	Obese class III	Underweight	Normal	Overweight	Obese class I	Obese class II	Obese class III
	n=383	n=9,685	n=9,785	n=3,519	n=886	n=436	n=134	n=4,087	n=5,307	n=1,684	n=373	n=178	n=249	n=5,598	n=4,478	n=1,835	n=513	n=258
**Mobility**																		
No problems	58.8	76.2	68.3	56.3	46.5	40.6	53.0	73.7	70.3	59.3	49.1	39.3	61.9	78.1	66.0	53.5	44.6	41.5
Slight problems	18.0	13.3	17.8	22.6	23.7	26.8	17.2	13.6	16.4	19.6	20.6	28.1	18.5	13.0	19.3	25.3	25.9	26.0
Moderate problems	10.4	6.7	9.5	13.8	18.1	17.7	17.2	7.8	8.9	13.4	17.4	16.9	6.8	5.9	10.2	14.1	18.5	18.2
Severe problems	7.8	2.9	3.5	6.3	9.9	12.6	9.0	3.9	3.5	6.6	11.5	12.9	7.2	2.2	3.6	6.0	8.8	12.4
Extreme problems	5.0	0.9	0.9	1.1	1.8	2.3	3.7	1.0	0.9	1.1	1.3	2.8	5.6	0.8	0.9	1.1	2.1	1.9
**Self-care**																		
No problems	78.3	91.9	90.6	86.4	80.0	77.3	73.1	90.3	90.4	85.9	77.8	73.0	81.1	93.0	90.8	86.9	81.7	80.2
Slight problems	8.9	4.4	5.7	8.0	12.4	12.4	11.2	5.5	6.0	8.0	15.3	14.0	7.6	3.6	5.3	8.0	10.3	11.2
Moderate problems	6.3	2.2	2.3	3.7	4.6	5.7	9.0	2.4	2.1	4.2	4.3	6.2	4.8	2.0	2.5	3.2	4.9	5.4
Severe problems	2.9	0.9	0.9	1.2	1.8	2.8	3.7	1.1	0.9	1.1	1.9	3.4	2.4	0.8	0.9	1.2	1.8	2.3
Extreme problems	3.7	0.7	0.6	0.8	1.1	1.8	3.0	0.7	0.6	0.8	0.8	3.4	4.0	0.6	0.6	0.7	1.4	0.8
**Usual activities**																		
No problems	58.5	75.0	70.9	60.9	51.6	49.1	57.5	74.3	73.4	63.7	54.4	49.4	59.0	75.5	67.9	58.3	49.5	48.8
Slight problems	18.0	15.0	18.2	24.1	25.7	26.6	18.7	15.2	16.6	22.2	22.3	25.3	17.7	14.8	20.1	25.8	28.3	27.5
Moderate problems	11.0	5.8	6.7	8.2	11.2	12.8	10.5	6.0	6.1	7.2	11.5	11.2	11.2	5.7	7.3	9.1	10.9	14.0
Severe problems	7.3	2.5	2.8	4.8	8.8	8.7	8.2	2.6	2.4	4.9	9.4	9.0	6.8	2.5	3.3	4.6	8.4	8.5
Extreme problems	5.2	1.7	1.5	2.1	2.7	2.8	5.2	1.9	1.5	2.0	2.4	5.1	5.2	1.6	1.4	2.1	2.9	1.2
**Pain/discomfort**																		
No problems	33.2	38.0	31.3	23.6	17.3	17.4	36.6	40.2	35.7	28.6	22.0	21.9	31.3	36.4	26.2	19.0	13.8	14.3
Slight problems	35.3	40.3	40.8	39.1	37.0	37.6	29.9	39.9	40.4	39.1	36.2	37.1	38.2	40.6	41.2	39.1	37.6	38.0
Moderate problems	21.9	17.6	23.1	29.6	33.1	28.7	22.4	15.9	20.5	26.0	29.0	27.0	21.7	18.9	26.3	32.9	36.1	29.8
Severe problems	8.6	3.7	4.5	7.1	11.5	15.4	9.7	3.7	3.2	5.8	11.5	12.9	8.0	3.7	5.9	8.4	11.5	17.1
Extreme problems	1.0	0.3	0.3	0.6	1.1	0.9	1.5	0.3	0.2	0.5	1.3	1.1	0.8	0.3	0.4	0.7	1.0	0.8
**Anxiety/depression**																		
No problems	57.4	63.8	65.0	60.3	55.0	45.6	64.9	69.3	69.6	67.0	63.0	47.8	53.4	59.7	59.5	54.2	49.1	44.2
Slight problems	28.7	28.8	27.6	29.9	29.9	40.6	20.2	24.7	24.7	24.9	23.3	38.8	33.3	31.8	31.1	34.4	34.7	41.9
Moderate problems	10.2	5.5	5.5	6.5	9.5	8.3	9.7	4.5	4.3	5.5	8.3	6.2	10.4	6.2	6.9	7.5	10.3	9.7
Severe problems	3.7	1.6	1.6	2.6	4.7	4.1	5.2	1.2	1.2	2.0	4.0	5.6	2.8	1.9	2.0	3.2	5.3	3.1
Extreme problems	0.0	0.4	0.3	0.6	0.9	1.4	0.0	0.3	0.2	0.5	1.3	1.7	0.0	0.5	0.4	0.7	0.6	1.2
**EQ VAS score (mean) [SD]**	70.2 [23.2]	78.7 [17.9]	76.9 [17.6]	72.5 [19.0]	67.2 [19.5]	66.4 [20.2]	65.9 [25.3]	78.4 [18.0]	77.8 [17.1]	74.0 [18.2]	68.1 [19.6]	64.8 [21.7]	72.5 [21.7]	79.0 [17.9]	75.9 [18.2]	71.1 [19.7]	66.6 [19.5]	67.4 [19.2]
**EQ VAS score (median) [IQR]**	80.0 [37.0]	83.0 [20.0]	80.0 [20.0]	75.0 [25.0]	70.0 [29.0]	70.0 [30.0]	75.0 [35.0]	82.0 [20.0]	80.0 [20.0]	80.0 [23.0]	70.0 [29.0]	70.0 [30.0]	80.0 [30.0]	83.0 [20.0]	80.0 [20.0]	75.0 [25.0]	70.0 [30.0]	70.0 [30.0]
**TTO index value (mean) [SD]**	0.855 [0.150]	0.907 [0.104]	0.899 [0.105]	0.872 [0.122]	0.839 [0.142]	0.828 [0.147]	0.847 [0.165]	0.908 [0.105]	0.907 [0.099]	0.882 [0.119]	0.846 [0.148]	0.825 [0.159]	0.859 [0.141]	0.906 [0.103]	0.889 [0.111]	0.864 [0.124]	0.835 [0.137]	0.831 [0.138]
**VAS index value (mean) [SD]**	71.8 [17.6]	77.9 [13.0]	76.6 [13.2]	73.2 [14.7]	69.1 [16.3]	67.8 [17.1]	71.4 [18.9]	78.2 [13.2]	77.8 [12.6]	74.6 [14.6]	70.1 [17.2]	67.8 [17.3]	71.9 [16.9]	77.6 [12.8]	75.2 [13.6]	71.9 [14.8]	68.4 [15.6]	67.8 [16.3]

Table S8 Problems in EQ-5D-5L dimensions (%), mean and median EQ VAS score, mean TTO index value, and mean VAS index value, total sample, by self-reported stress and self-reported sickness (30–69 years), and number of diagnosed diseases (30–104 years)

EQ-5D-5L dimension	Stress (30-69 years)					Sick for more than 29 continuous days in the past 12 months (30-69 years)							Number of diseases diagnosed by a physician (30-104 years)			
	Quite a lot/very much (com-bined)	Not at all	To some extent	Quite a lot	Very much	No, I have not been sick for a continuous period of 29 days or more	Yes, for problems with my back, neck, joints or muscles	Yes, for exhaustion, fatigue, depression, stress	Yes, for other mental health problems (including insomnia)	Yes, for cardio-vascular disease, stroke	Yes for accident, injury	Yes for other reasons	One	Two	Three	Four or more
	n=1,676	n=6,142	n=6,170	n=1,285	n=391	n=9,151	n=317	n=455	n=120	n=60	n=187	n=344	n=8,182	n=2,349	n=383	n=79
**Mobility**																
No problems	64.9	85.4	78.4	67.7	55.5	87.0	42.6	71.4	54.2	43.3	62.0	62.5	59.3	44.9	33.2	16.5
Slight problems	18.5	10.0	14.4	17.6	21.5	9.9	28.1	15.8	23.3	28.3	19.8	21.5	21.4	25.5	21.4	20.3
Moderate problems	11.0	3.2	5.0	10.3	13.6	2.5	20.8	10.8	17.5	16.7	11.2	10.5	12.6	18.5	23.2	25.3
Severe problems	5.0	1.1	2.0	4.0	8.4	0.6	7.9	1.8	3.3	8.3	5.4	5.2	5.5	9.3	18.5	25.3
Extreme problems	0.6	0.3	0.2	0.5	1.0	0.1	0.6	0.2	1.7	3.3	1.6	0.3	1.2	1.8	3.7	12.7
**Self-care**																
No problems	84.7	96.9	94.3	86.5	79.0	97.6	72.9	87.7	75.0	81.7	86.6	85.8	87.1	79.6	61.9	41.8
Slight problems	9.8	2.2	4.2	9.2	11.8	2.1	18.0	8.4	16.7	13.3	9.1	10.8	7.5	11.3	17.8	27.9
Moderate problems	4.1	0.6	1.2	3.2	6.9	0.3	7.9	3.3	5.8	3.3	3.2	2.9	3.3	5.5	12.8	11.4
Severe problems	1.2	0.2	0.3	1.0	1.8	0.1	1.3	0.0	2.5	0.0	1.1	0.6	1.3	2.6	5.5	8.9
Extreme problems	0.2	0.1	0.1	0.2	0.5	0.0	0.0	0.0	0.0	1.7	0.0	0.0	0.9	1.1	2.1	10.1
**Usual activities**																
No problems	49.6	86.4	75.0	53.2	37.9	85.5	28.7	39.3	25.0	35.0	48.1	54.1	62.0	49.3	32.4	19.0
Slight problems	24.2	10.3	17.7	24.8	22.0	11.9	32.5	29.2	27.5	28.3	28.3	25.3	22.0	28.7	27.9	26.6
Moderate problems	13.7	2.0	4.6	12.9	16.1	1.9	23.3	18.7	23.3	18.3	12.8	9.3	9.4	11.9	17.0	19.0
Severe problems	10.1	0.9	2.2	7.3	19.2	0.6	12.6	11.0	18.3	11.7	6.4	7.0	4.5	7.1	15.7	21.5
Extreme problems	2.5	0.4	0.4	1.7	4.9	0.1	2.8	1.8	5.8	6.7	4.3	4.4	2.1	3.0	7.1	13.9
**Pain/discomfort**																
No problems	19.5	45.8	31.9	21.3	13.8	42.2	4.1	21.8	16.7	10.0	13.4	20.6	24.8	16.7	8.4	11.4
Slight problems	32.9	39.2	43.7	35.3	25.1	42.2	22.1	36.3	23.3	36.7	38.5	35.5	41.0	36.9	29.0	19.0
Moderate problems	32.0	12.9	19.8	30.9	35.6	13.7	46.1	29.5	39.2	38.3	34.2	32.3	27.2	35.2	41.0	39.2
Severe problems	13.7	1.9	4.4	11.5	20.7	1.9	26.5	12.1	17.5	11.7	12.3	10.5	6.5	10.6	17.5	25.3
Extreme problems	1.9	0.1	0.2	1.0	4.9	0.1	1.3	0.4	3.3	3.3	1.6	1.2	0.5	0.6	4.2	5.1
**Anxiety/depression**																
No problems	13.1	89.1	47.5	14.6	8.2	65.9	37.9	17.6	3.3	45.0	55.1	48.6	60.1	51.5	25.6	25.3
Slight problems	38.8	10.3	45.4	43.4	23.5	28.5	39.4	42.2	34.2	31.7	30.5	36.3	29.5	33.1	35.3	32.9
Moderate problems	29.2	0.4	6.1	30.4	25.3	4.5	15.1	22.9	26.7	11.7	11.2	12.5	7.1	10.6	26.4	21.5
Severe problems	15.2	0.2	0.9	10.4	31.0	1.1	6.0	14.1	24.2	6.7	2.7	1.7	2.6	4.3	9.7	16.5
Extreme problems	3.8	0.1	0.1	1.3	12.0	0.1	1.6	3.3	11.7	5.0	0.5	0.9	0.7	0.5	3.1	3.8
**EQ VAS score (mean) [SD]**	61.7 (21.4)	84.5 (13.6)	77.3 (15.5)	64.8 (19.9)	51.5 (23.1)	81.6 (13.9)	62.2 (21.6)	63.7 (20.8)	54.6 (21.2)	65.3 (20.6)	70.5 (18.3)	68.6 (21.8)	72.8 (19.2)	67.0 (19.8)	54.9 (22.0)	51.2 (23.3)
**EQ VAS score (median) [IQR]**	65.0 [30.0]	90.0 [15.0]	80.0 [20.0]	70.0 [30.0]	50.0 [35.0]	85.0 [15.0]	65.0 [34.0]	70.0 [30.0]	50.0 [35.0]	70.0 [30.0]	75.0 [23.0]	75.0 [35.0]	77.0 [28.0]	70.0 [30.0]	60.0 [35.0]	50.0 [35.0]
**TTO index value (mean) [SD]**	0.795 (0.159)	0.943 (0.061)	0.907 (0.087)	0.820 (0.139)	0.710 (0.187)	0.933 (0.066)	0.780 (0.147)	0.803 (0.148)	0.714 (0.171)	0.797 (0.166)	0.848 (0.129)	0.855 (0.124)	0.875 (0.121)	0.839 (0.136)	0.748 (0.179)	0.688 (0.189)
**VAS index value (mean) [SD]**	64.0 (16.9)	82.8 (8.4)	77.2 (11.2)	66.6 (15.2)	55.3 (19.1)	81.0 (9.0)	62.2 (16.1)	65.3 (16.1)	55.4 (17.5)	64.0 (18.6)	70.0 (15.1)	70.9 (15.0)	73.6 (14.6)	69.0 (15.8)	58.8 (19.0)	52.2 (20.4)

Comparing proportions of any problems (chi-square tests) and mean EQ VAS scores, TTO index values and VAS index values (independent t-tests) between groups with a specific condition with groups without having the specific condition and across different numbers of diseases showed statistically significant differences in all comparisons. Slight, moderate, severe and extreme problems were collapsed into any problems for the chi-square test.

Table S9 Proportion of self-rated health (SRH) levels by sex, by age group (n=25,867)

Self-rated health	Age group (years)													
	**Total sample**	**30-34**	**35-39**	**40-44**	**45-49**	**50-54**	**55-59**	**60-64**	**65-69**	**70-74**	**75-79**	**80-84**	**85-89**	**90-94**	**95-104**
	n=25,867	n=1,127	n=1,202	n=1,473	n=1,722	n=1,824	n=1,917	n=2,144	n=2,647	n=4,489	n=2,972	n=1,723	n=1,762	n=703	n=162
**Total sample**															
Very good	15.7	25.6	25.0	23.0	22.2	18.9	18.9	16.5	17.4	15.2	10.6	7.1	5.0	3.6	1.8
Good	48.9	55.2	54.2	55.1	53.5	52.1	49.7	51.6	52.7	51.1	46.0	40.7	34.7	29.9	33.3
Neither good nor bad	28.5	13.4	16.6	16.8	19.0	21.5	25.2	25.9	25.1	28.3	36.3	42.2	47.3	53.1	46.3
Bad	5.1	4.1	3.0	4.1	4.2	6.1	4.7	4.6	4.1	3.7	5.3	7.3	9.5	9.2	11.7
Very bad	0.8	0.7	0.7	0.7	1.0	0.7	1.0	0.7	0.3	0.9	0.9	1.5	2.0	3.1	5.6
Missing	0.7	0.9	0.6	0.3	0.1	0.8	0.5	0.8	0.4	0.8	0.9	1.2	1.4	1.1	1.2
**Men**															
Very good	15.7	28.4	29.3	26.4	23.1	18.9	17.1	15.5	16.8	15.2	11.8	7.1	5.8	4.4	0.0
Good	50.2	58.1	56.7	56.1	57.1	54.7	54.5	52.7	53.7	51.8	46.1	42.8	36.5	32.7	35.9
Neither good nor bad	27.5	8.0	10.8	13.7	15.9	20.2	22.1	26.0	24.8	27.5	35.2	40.7	44.6	50.3	46.9
Bad	5.0	3.2	2.0	2.9	3.2	4.9	4.6	4.6	4.0	4.1	5.4	7.0	10.0	8.8	14.1
Very bad	1.0	0.9	0.8	0.6	0.7	0.6	1.1	0.6	0.4	0.8	0.9	1.5	2.0	3.3	3.1
Missing	0.6	1.4	0.4	0.2	0.1	0.8	0.6	0.6	0.3	0.6	0.6	0.8	1.2	0.6	0.0
**Women**															
Very good	15.7	23.9	22.0	20.4	21.5	18.8	20.3	17.3	17.9	15.2	9.3	7.0	4.0	2.6	3.1
Good	47.8	53.3	52.4	54.4	50.7	50.2	46.0	50.7	51.9	50.3	46.0	38.7	32.2	26.8	31.6
Neither good or bad	29.4	16.8	20.7	19.1	21.4	22.5	27.6	25.8	25.3	29.2	37.4	43.7	50.9	56.0	45.9
Bad	5.2	4.6	3.7	4.9	4.9	7.0	4.7	4.5	4.1	3.3	5.2	7.6	9.0	9.7	10.2
Very bad	1.0	0.7	0.6	0.7	1.3	0.7	1.0	0.8	0.2	1.1	1.0	1.5	2.1	3.0	7.1
Missing	0.8	0.6	0.7	0.5	0.1	0.8	0.4	0.9	0.5	0.9	1.2	1.5	1.7	1.8	2.0

Table S10 Problems in EQ-5D-5L dimensions (%), mean and median EQ VAS score, mean TTO index value, and mean VAS index value, total sample, by self-rated health (SRH) level, 30–104 years (n=25,677)

**EQ-5D-5L dimension**	**Self-rated health (SRH)**				
	**Very good**	**Good**	**Neither good nor bad**	**Bad**	**Very bad**
	n=4,064	n=12,650	n=7,378	n=1,323	n=262
**Mobility**					
No problems	96.5	81.0	40.0	17.8	12.2
Slight problems	2.7	14.6	29.5	19.0	10.7
Moderate problems	0.5	3.5	21.7	28.0	17.6
Severe problems	0.2	0.7	7.2	29.0	41.6
Extreme problems	0.2	0.3	1.6	6.2	17.9
**Self-care**					
No problems	99.5	96.9	79.9	48.7	27.5
Slight problems	0.3	2.2	12.4	22.0	16.0
Moderate problems	0.1	0.5	5.2	16.2	19.1
Severe problems	0.0	0.2	1.5	7.9	19.5
Extreme problems	0.0	0.2	1.0	5.3	17.9
**Usual activities**					
No problems	97.6	84.7	40.2	10.3	4.6
Slight problems	1.9	12.6	35.9	24.0	6.5
Moderate problems	0.2	1.7	16.2	26.1	16.0
Severe problems	0.2	0.6	5.3	26.8	36.3
Extreme problems	0.1	0.4	2.4	12.9	36.6
**Pain/discomfort**					
No problems	71.1	36.0	9.4	4.4	5.3
Slight problems	25.3	49.8	36.7	13.4	8.0
Moderate problems	3.4	13.1	45.0	44.7	25.6
Severe problems	0.2	1.0	8.6	35.4	41.2
Extreme problems	0.02	0.05	0.4	2.1	19.8
**Anxiety/depression**					
No problems	89.0	71.5	44.0	15.3	14.1
Slight problems	10.3	25.8	42.7	37.7	21.0
Moderate problems	0.5	2.2	10.7	27.7	24.4
Severe problems	0.1	0.3	2.3	16.5	26.0
Extreme problems	0.02	0.1	0.3	2.9	14.5
**EQ VAS score (mean) [SD]**	92.0 [8.7]	82.2 [11.3]	64.3 [15.6]	41.5 [16.9]	24.2 [20.3]
**EQ VAS score (median) [IQR]**	95.0 [8.0]	85.0 [15.0]	67.0 [25.0]	40.0 [20.0]	20.0 [23.0]
**TTO index value (mean) [SD]**	0.964 [0.029]	0.935 [0.056]	0.833 [0.109]	0.667 [0.140]	0.535 [0.161]
**VAS index value (mean) [SD]**	86.2 [4.8]	81.1 [8.0]	67.6 [12.7]	49.3 [14.0]	36.1 [16.3]

Table S11 Odds Ratios (OR) (multivariable logistic regression) for reporting any problems on the EQ-5D-5L dimensions and respondent's sex, age, educational level and income, 30–104 years (n=25,867)

	Mobility			Self-care			Usual activities			Pain/discomfort			Anxiety/depression		
	OR	95% CI	P-value	OR	95% CI	P-value	OR	95% CI	P-value	OR	95% CI	P-value	OR	95% CI	P-value
**Sex[a]**															
Women	**1.07**	**1.01-1.14**	**0.0261**	**0.81**	**0.74-0.88**	**<.0001**	**1.10**	**1.04-1.17**	**0.0011**	**1.37**	**1.29-1.45**	**<.0001**	**1.38**	**1.31-1.46**	**<.0001**
**Age group[b]**															
35-39	1.00	0.77-1.30	0.9943	**0.49**	**0.28-0.83**	**0.0089**	0.95	0.77-1.18	0.6343	1.05	0.89-1.24	0.5641	0.85	0.72-1.00	0.0545
40-44	**1.34**	**1.04-1.71**	**0.0213**	1.29	0.85-1.95	0.2261	1.18	0.97-1.45	0.1027	**1.45**	**1.24-1.70**	**<.0001**	0.89	0.76-1.04	0.1390
45-49	**1.73**	**1.37-2.18**	**<.0001**	1.47	0.99-2.17	0.0544	**1.35**	**1.11-1.64**	**0.0023**	**1.56**	**1.34-1.82**	**<.0001**	**0.75**	**0.64-0.87**	**0.0002**
50-54	**2.27**	**1.82-2.84**	**<.0001**	**2.18**	**1.51-3.14**	**<.0001**	**1.46**	**1.21-1.76**	**<.0001**	**1.93**	**1.65-2.25**	**<.0001**	**0.78**	**0.67-0.91**	**0.0014**
55-59	**2.81**	**2.26-3.49**	**<.0001**	**2.59**	**1.81-3.70**	**<.0001**	**1.72**	**1.43-2.07**	**<.0001**	**2.22**	**1.90-2.59**	**<.0001**	**0.59**	**0.51-0.69**	**<.0001**
60-64	**3.20**	**2.59-3.96**	**<.0001**	**2.22**	**1.56-3.17**	**<.0001**	**1.75**	**1.46-2.10**	**<.0001**	**2.30**	**1.98-2.68**	**<.0001**	**0.50**	**0.43-0.58**	**<.0001**
65-69	**3.04**	**2.47-3.75**	**<.0001**	**1.82**	**1.28-2.58**	**0.0008**	**1.33**	**1.12-1.59**	**0.0015**	**2.16**	**1.87-2.50**	**<.0001**	**0.37**	**0.32-0.43**	**<.0001**
70-74	**3.26**	**2.67-3.99**	**<.0001**	**1.98**	**1.42-2.76**	**<.0001**	**1.26**	**1.06-1.49**	**0.0078**	**1.78**	**1.55-2.04**	**<.0001**	**0.38**	**0.33-0.44**	**<.0001**
75-79	**4.49**	**3.66-5.51**	**<.0001**	**2.46**	**1.76-3.44**	**<.0001**	**1.74**	**1.47-2.07**	**<.0001**	**2.14**	**1.84-2.48**	**<.0001**	**0.40**	**0.34-0.46**	**<.0001**
80-84	**7.07**	**5.71-8.77**	**<.0001**	**4.04**	**2.88-5.69**	**<.0001**	**2.71**	**2.26-3.26**	**<.0001**	**2.57**	**2.17-3.04**	**<.0001**	**0.43**	**0.37-0.51**	**<.0001**
85-89	**12.88**	**10.36-16.01**	**<.0001**	**6.37**	**4.55-8.92**	**<.0001**	**4.31**	**3.59-5.18**	**<.0001**	**3.04**	**2.56-3.61**	**<.0001**	**0.57**	**0.49-0.67**	**<.0001**
90-94	**20.75**	**15.93-27.02**	**<.0001**	**13.28**	**9.33-18.89**	**<.0001**	**7.40**	**5.89-9.29**	**<.0001**	**3.75**	**2.96-4.75**	**<.0001**	**0.61**	**0.50-0.74**	**<.0001**
95-104	**42.19**	**25.13-70.83**	**<.0001**	**32.67**	**20.65-51.67**	**<.0001**	**12.13**	**7.93-18.54**	**<.0001**	**3.81**	**2.43-5.95**	**<.0001**	0.89	0.63-1.25	0.4929
**Educational level[c]**															
Medium	**0.91**	**0.84-0.97**	**0.0077**	**0.90**	**0.81-0.99**	**0.0385**	0.97	0.91-1.05	0.4657	1.00	0.93-1.08	0.9450	0.99	0.92-1.06	0.7485
High	**0.72**	**0.66-0.78**	**<.0001**	**0.74**	**0.65-0.84**	**<.0001**	**0.76**	**0.70-0.82**	**<.0001**	**0.71**	**0.66-0.78**	**<.0001**	1.00	0.92-1.08	0.9524
Missing	***1.54***	***1.05-2.25***	***0.0271***	***1.95***	***1.28-2.98***	***0.0019***	***1.73***	***1.20-2.50***	***0.0034***	*0.84*	*0.57-1.22*	*0.3514*	*1.27*	*0.90-1.80*	*0.1793*
**Income quintile[d]**															
Second	1.03	0.95-1.12	0.4372	0.95	0.85-1.06	0.3275	0.98	0.90-1.06	0.6189	1.06	0.97-1.16	0.2246	**0.92**	**0.85-0.99**	**0.033**
Third	**0.78**	**0.71-0.85**	**<.0001**	**0.67**	**0.59-0.76**	**<.0001**	**0.73**	**0.67-0.80**	**<.0001**	**0.85**	**0.78-0.93**	**0.0005**	**0.70**	**0.64-0.76**	**<.0001**
Fourth	**0.59**	**0.53-0.65**	**<.0001**	**0.45**	**0.38-0.53**	**<.0001**	**0.51**	**0.46-0.56**	**<.0001**	**0.73**	**0.66-0.80**	**<.0001**	**0.53**	**0.49-0.58**	**<.0001**
Fifth (highest)	**0.46**	**0.41-0.51**	**<.0001**	**0.32**	**0.27-0.38**	**<.0001**	**0.41**	**0.37-0.45**	**<.0001**	**0.59**	**0.53-0.65**	**<.0001**	**0.44**	**0.40-0.48**	**<.0001**
Missing	*0.88*	*0.48-1.61*	*0.6685*	0.41	0.12-1.34	0.1383	***0.41***	***0.21-0.77***	***0.0063***	***0.43***	***0.26-0.71***	***0.0009***	**0.32**	**0.19-0.54**	**<.0001**
**N**	25,867			25,867			25,867			25,867			25,867		

Reference groups: [a]Men; [b]30–34 years; [c]Low educational level; [d]First quintile (lowest)
Statistically significant estimates are shown in bold (<0.05)

Women were significantly more likely than men to report problems with mobility, usual activities, pain/discomfort and anxiety/depression and less likely to report problems with self-care. Having high or medium education resulted in statistically significantly lower OR to report problems with mobility and self-care compared to those with low education. Those with high education had significantly lower OR to report problems with usual activities and pain/discomfort. There was no significant difference between educational groups for reporting problems with anxiety/depression. In all dimensions, those in the fifth (highest), fourth and third income quintiles had significantly lower OR to report problems than those in the first (lowest) income quintile. There were no significant difference between those in the next to lowest and lowest income quintiles in any dimension, expect that those in the next to the lowest had significantly higher OR to report problems with anxiety/depression than those in the lowest quintile.

Table S12 Odds Ratios (OR) (multivariable logistic regression) for reporting any problems on the EQ-5D-5L dimensions and respondent's sex, age, educational level, income and economic activity, 30–64 years (n=11,409)

	**Mobility**			**Self-care**			**Usual activities**			**Pain/discomfort**			**Anxiety/depression**		
	**OR**	**95% CI**	**P-value**	**OR**	**95% CI**	**P-value**	**OR**	**95% CI**	**P-value**	**OR**	**95% CI**	**P-value**	**OR**	**95% CI**	**P-value**
**Sex[a]**															
Men	1			1			1			1			1		
Women	1.1	0.98-1.22	0.0980	**0.79**	**0.65-0.94**	**0.0092**	**1.20**	**1.09-1.33**	**0.0004**	**1.36**	**1.25-1.47**	**<0.0001**	**1.36**	**1.26-1.48**	**<0.0001**
**Age group[b]**															
35-39	1.06	0.81-1.39	0.6917	**0.52**	**0.30-0.91**	**0.0219**	0.99	0.79-1.23	0.9179	1.05	0.89-1.24	0.5591	0.86	0.73-1.02	0.0735
40-44	**1.39**	**1.08-1.79**	**0.0110**	1.30	0.84-1.99	0.2356	1.19	0.96-1.46	0.1096	**1.42**	**1.21-1.67**	**<.0001**	0.88	0.75-1.03	0.1153
45-49	**1.81**	**1.43-2.30**	**<.0001**	1.47	0.98-2.22	0.0646	**1.36**	**1.11-1.67**	**0.0026**	**1.52**	**1.30-1.79**	**<.0001**	**0.73**	**0.63-0.86**	**0.0001**
50-54	**2.31**	**1.83-2.92**	**<.0001**	**2.02**	**1.37-2.97**	**0.0004**	**1.41**	**1.16-1.72**	**0.0007**	**1.86**	**1.59-2.18**	**<.0001**	**0.76**	**0.65-0.89**	**0.0005**
55-59	**2.80**	**2.23-3.52**	**<.0001**	**2.28**	**1.56-3.33**	**<.0001**	**1.63**	**1.34-1.98**	**<.0001**	**2.10**	**1.79-2.46**	**<.0001**	**0.56**	**0.47-0.65**	**<.0001**
60-64	**2.84**	**2.26-3.56**	**<.0001**	**1.50**	**1.02-2.19**	**0.0387**	**1.42**	**1.17-1.73**	**0.0004**	**2.08**	**1.77-2.44**	**<.0001**	**0.45**	**0.38-0.52**	**<.0001**
**Educational level[c]**															
Medium	**0.84**	**0.71-1.00**	**0.0460**	0.82	0.63-1.05	0.1176	0.93	0.79-1.10	0.3914	0.90	0.77-1.06	0.1921	1.00	0.86-1.16	0.9615
High	**0.67**	**0.56-0.80**	**<.0001**	**0.74**	**0.55-0.98**	**0.0346**	**0.74**	**0.62-0.88**	**0.0005**	**0.62**	**0.52-0.72**	**<.0001**	1.06	0.90-1.23	0.4964
Missing	*1.74*	*0.87-3.50*	*0.1184*	*1.38*	*0.51-3.73*	*0.5320*	*1.16*	*0.56-2.39*	*0.6939*	*0.57*	*0.3-1.1*	*0.0942*	*0.99*	*0.50-1.94*	*0.9733*
**Income quintile[d]**															
Second	**1.35**	**1.09-1.67**	**0.0067**	**1.43**	**1.06-1.91**	**0.0177**	**1.44**	**1.17-1.77**	**0.0006**	1.18	0.96-1.46	0.1244	1.11	0.92-1.35	0.2767
Third	1.01	0.85-1.21	0.9094	1.13	0.87-1.49	0.3647	1.08	0.91-1.28	0.3834	1.09	0.93-1.28	0.2881	0.92	0.79-1.07	0.2792
Fourth	**0.76**	**0.64-0.91**	**0.0025**	**0.72**	**0.54-0.97**	**0.0279**	**0.75**	**0.63-0.88**	**0.0007**	0.92	0.79-1.07	0.2845	**0.73**	**0.63-0.84**	**<.0001**
Fifth (highest)	**0.53**	**0.44-0.64**	**<.0001**	**0.44**	**0.32-0.62**	**<.0001**	**0.55**	**0.46-0.66**	**<.0001**	**0.68**	**0.59-0.80**	**<.0001**	**0.55**	**0.47-0.64**	**<.0001**
Missing	*0.92*	*0.48-1.77*	*0.7976*	*0.48*	*0.14-1.70*	*0.2572*	***0.43***	***0.21-0.88***	***0.0200***	***0.47***	***0.27-0.81***	***0.0065***	***0.32***	***0.18-0.57***	***0.0001***
**Economic activity[e]**															
Studying/ parental leave	1.14	0.83-1.55	0.4177	0.96	0.49-1.85	0.8907	1.10	0.84-1.43	0.5073	0.88	0.71-1.09	0.2249	0.98	0.80-1.22	0.8845
Unemployed	**2.68**	**2.13-3.37**	**<.0001**	**4.02**	**2.91-5.56**	**<.0001**	**2.53**	**2.03-3.15**	**<.0001**	**1.37**	**1.08-1.73**	**0.0092**	**3.01**	**2.40-3.78**	**<.0001**
Retired	1.35	0.89-2.05	0.1567	1.36	0.58-3.18	0.4823	1.29	0.84-1.97	0.2453	0.97	0.65-1.45	0.8814	0.67	0.43-1.05	0.0775
Sick leave	**3.91**	**3.30-4.63**	**<.0001**	**8.61**	**6.82-10.87**	**<.0001**	**5.80**	**4.90-6.87**	**<.0001**	**2.37**	**1.94-2.9**	**<.0001**	**2.72**	**2.30-3.20**	**<.0001**
Missing	*1.39*	*0.86-2.27*	*0.1826*	***2.60***	***1.28-5.26***	***0.0082***	*1.33*	*0.84-2.09*	*0.2264*	*1.02*	*0.68-1.53*	*0.9217*	*1.41*	*0.95-2.08*	*0.0896*
**N**	11,409			11,409			11,409			11,409			11,409		

Reference groups: [a]Men; [b]30–34 years; [c]Low educational level; [d]First quintile (lowest); [e]Employed
Statistically significant estimates are shown in bold (<0.05)

Unemployed respondents, and those on sick leave, had significantly higher OR to report problems in all dimensions than those who were employed.

Table S13 Ordinary Least Square (OLS) regression on mean EQ VAS score adjusted for sex, age, educational level, income and economic activity, 30–64 years (n=10,800)

**Variable**	**EQ VAS score**											
	**Model 1**			**Model 2**			**Model 3**			**Model 4**		
	**Estimate**	**RSE**	**P-value**	**Estimate**	**RSE**	**P-value**	**Estimate**	**RSE**	**P-value**	**Estimate**	**RSE**	**P-value**
**Intercept**	**76.500**	**0.842**	**<.0001**	**73.831**	**0.772**	**<.0001**	**72.038**	**0.982**	**<.0001**	**79.051**	**0.968**	**<.0001**
**Sex**[a]												
Women	**-2.772**	**0.329**	**<.0001**	**-1.113**	**0.337**	**0.0010**	**-1.462**	**0.346**	**<.0001**	**-1.075**	**0.332**	**0.0012**
**Age group**[b]												
35-39	-0.487	0.671	0.4679	-1.277	0.679	0.0599	-1.246	0.676	0.0656	-1.191	0.661	0.0714
40-44	-0.549	0.655	0.4017	**-2.470**	**0.661**	**0.0002**	**-2.285**	**0.661**	**0.0005**	**-1.650**	**0.638**	**0.0098**
45-49	-0.754	0.642	0.2404	**-2.818**	**0.646**	**<.0001**	**-2.540**	**0.648**	**<.0001**	**-1.900**	**0.633**	**0.0027**
50-54	-1.005	0.647	0.1207	**-3.080**	**0.646**	**<.0001**	**-2.662**	**0.651**	**<.0001**	**-1.877**	**0.630**	**0.0029**
55-59	-0.485	0.647	0.4538	**-2.619**	**0.646**	**<.0001**	**-2.146**	**0.649**	**0.0010**	-1.083	0.634	0.0875
60-64	0.457	0.625	0.4651	**-1.366**	**0.618**	**0.0271**	-0.803	0.628	0.2010	**1.344**	**0.630**	**0.0329**
**Educational level**[c]												
Medium	**2.800**	**0.734**	**0.0001**				1.336	0.709	0.0594	0.423	0.665	0.5251
High	**5.492**	**0.730**	**<.0001**				**3.015**	**0.716**	**<.0001**	**1.360**	**0.676**	**0.0441**
Missing	*-0.011*	*0.033*	*0.7448*				*-0.012*	*0.032*	*0.7028*	*0.021*	*0.033*	*0.5226*
**Income** (individual) (thousand SEK)[d]												
Second quintile				-0.488	1.039	0.6384	-0.445	1.035	0.6671	**-2.176**	**0.963**	**0.0239**
Third quintile				**5.956**	**0.746**	**<.0001**	**5.908**	**0.743**	**<.0001**	0.495	0.730	0.4981
Fourth quintile				**8.849**	**0.689**	**<.0001**	**8.530**	**0.685**	**<.0001**	**2.227**	**0.680**	**0.0011**
Fifth quintile				**10.309**	**0.691**	**<.0001**	**9.627**	**0.694**	**<.0001**	**3.393**	**0.690**	**<.0001**
Missing				***0.075***	***0.026***	***0.0036***	***0.081***	***0.025***	***0.0011***	***0.055***	***0.026***	***0.0332***
**Economic activity**[e]												
Studying/parental leave										-1.577	0.950	0.0970
Unemployed										**-10.629**	**1.264**	**<.0001**
Retired										-0.765	1.329	0.5649
Sick leave										**-19.398**	**0.937**	**<.0001**
Missing										*-2.172*	*1.837*	*0.2370*
**Adjusted R^2**	0.0144			0.0477			0.0506			0.1249		
**RMSE**	17.00			16.71			16.69			16.02		
**N**	10,800			10,800			10,800			10,800		

Reference groups: [a]Men; [b]30–34 years; [c]Low educational level; [d]First quintile (lowest); [e]Employed
RSE: Robust Standard Error. Statistically significant estimates are shown in bold ((<0.05)

Table S14 Ordinary Least Square (OLS) regression on mean EQ VAS score by health-related behaviours and diseases diagnosed by a physician, adjusted for sex, age, educational level and income, 30–104 years (n=23,899)

Variable	EQ VAS score																	
	Model 1			Model 2			Model 3			Model 4			Model 5			Model 6		
	Estimate	RSE	P-value	Estimate	RSE	P-value	Estimate	RSE	P-value	Estimate	RSE	P-value	Estimate	RSE	P-value	Estimate	RSE	P-value
**Intercept**	**84.371**	**0.507**	**<.0001**	**78.741**	**0.651**	**<.0001**	**81.782**	**0.634**	**<.0001**	**76.074**	**0.628**	**<.0001**	**82.666**	**0.453**	**<.0001**	**77.278**	**0.603**	**77.278**
**Sex[a]**																		
Women	**-1.852**	**0.224**	**<.0001**	**-0.825**	**0.235**	**0.0004**	**-1.383**	**0.223**	**<.0001**	-0.388	0.236	0.1008	**-0.965**	**0.220**	**<.0001**	-0.106	0.232	0.6465
**Age group[b]**																		
35-39	-0.630	0.676	0.3512	-1.187	0.673	0.0778	-0.741	0.644	0.2497	-1.221	0.645	0.0585	-1.005	0.618	0.1039	**-1.412**	**0.621**	**0.0230**
40-44	-0.688	0.655	0.2940	**-1.853**	**0.653**	**0.0046**	-0.806	0.624	0.1965	**-1.870**	**0.626**	**0.0028**	**-1.188**	**0.597**	**0.0466**	**-2.102**	**0.602**	**0.0005**
45-49	-0.963	0.635	0.1294	**-2.079**	**0.633**	**0.0010**	-0.632	0.609	0.2993	**-1.667**	**0.611**	**0.0064**	**-1.278**	**0.582**	**0.0282**	**-2.147**	**0.588**	**0.0003**
50-54	**-1.732**	**0.638**	**0.0066**	**-2.591**	**0.635**	**<.0001**	-0.739	0.610	0.2254	**-1.571**	**0.612**	**0.0103**	**-1.608**	**0.584**	**0.0059**	**-2.274**	**0.589**	**0.0001**
55-59	-0.976	0.638	0.1264	**-1.807**	**0.635**	**0.0044**	0.313	0.612	0.6087	-0.461	0.612	0.4514	-0.585	0.584	0.3166	**-1.199**	**0.587**	**0.0411**
60-64	-0.346	0.614	0.5731	-0.774	0.612	0.2060	**1.890**	**0.591**	**0.0014**	**1.456**	**0.593**	**0.0141**	0.373	0.566	0.5096	0.093	0.571	0.8713
65-69	-0.107	0.588	0.8553	0.260	0.588	0.6584	**3.389**	**0.573**	**<.0001**	**3.653**	**0.576**	**<.0001**	**1.397**	**0.552**	**0.0114**	**1.742**	**0.557**	**0.0018**
70-74	**-1.349**	**0.551**	**0.0143**	0.319	0.558	0.5678	**2.529**	**0.539**	**<.0001**	**4.009**	**0.548**	**<.0001**	0.607	0.519	0.2422	**1.993**	**0.530**	**0.0002**
75-79	**-4.576**	**0.590**	**<.0001**	**-2.148**	**0.606**	**0.0004**	-0.852	0.583	0.1440	**1.381**	**0.601**	**0.0216**	**-2.784**	**0.566**	**<.0001**	-0.741	0.584	0.2044
80-84	**-7.718**	**0.685**	**<.0001**	**-4.987**	**0.704**	**<.0001**	**-5.035**	**0.694**	**<.0001**	**-2.399**	**0.715**	**0.0008**	**-6.804**	**0.670**	**<.0001**	**-4.400**	**0.691**	**<.0001**
85-89	**-11.461**	**0.715**	**<.0001**	**-8.556**	**0.737**	**<.0001**	**-10.210**	**0.727**	**<.0001**	**-7.358**	**0.750**	**<.0001**	**-12.059**	**0.709**	**<.0001**	**-9.451**	**0.733**	**<.0001**
90-94	**-13.788**	**1.006**	**<.0001**	**-10.762**	**1.025**	**<.0001**	**-15.356**	**1.044**	**<.0001**	**-12.291**	**1.065**	**<.0001**	**-17.081**	**1.023**	**<.0001**	**-14.286**	**1.043**	**<.0001**
95-104	**-16.122**	**1.963**	**<.0001**	**-12.984**	**1.975**	**<.0001**	**-20.509**	**2.113**	**<.0001**	**-17.253**	**2.127**	**<.0001**	**-21.284**	**2.109**	**<.0001**	**-18.324**	**2.118**	**<.0001**
**Educational level[c]**																		
Medium				0.551	0.325	0.0902				**0.908**	**0.329**	**0.0058**				**0.853**	**0.322**	**0.0081**
High				**1.611**	**0.349**	**<.0001**				**1.722**	**0.351**	**<.0001**				**1.749**	**0.344**	**<.0001**
Missing				*0.007*	*0.018*	*0.7026*				*0.004*	*0.018*	*0.8424*				*0.006*	*0.018*	*0.7405*
**Income** (individual) (thousand SEK)[d]																		
Second quintile				0.434	0.401	0.2785				**0.991**	**0.408**	**0.0151**				**1.001**	**0.398**	**0.0118**
Third quintile				**3.238**	**0.392**	**<.0001**				**3.803**	**0.396**	**<.0001**				**3.431**	**0.386**	**<.0001**
Fourth quintile				**5.387**	**0.407**	**<.0001**				**5.604**	**0.407**	**<.0001**				**4.894**	**0.396**	**<.0001**
Fifth quintile				**6.859**	**0.421**	**<.0001**				**6.502**	**0.420**	**<.0001**				**5.783**	**0.410**	**<.0001**
Missing				***0.092***	***0.023***	***<.0001***				***0.067***	***0.022***	***0.0027***				***0.051***	***0.021***	***0.0168***
**Tobacco and alcohol**																		
Daily smoking[e]	**-3.322**	**0.535**	**<.0001**	**-2.234**	**0.525**	**<.0001**												
Risk consumption of alcohol[f]	-0.583	0.384	0.1288	**-0.910**	**0.379**	**0.0163**												
**Physical exercise[g]**																		
Less than 150 minutes/week	**-7.356**	**0.253**	**<.0001**	**-6.650**	**0.254**	**<.0001**												
**Sitting[h]**																		
Sitting for 10 or more hours/day	**-10.235**	**0.409**	**<.0001**	**-10.480**	**0.403**	**<.0001**												
**Diseases diagnosed by a physician[i]**																		
Asthma													**-3.343**	**0.479**	**<.0001**	**-3.288**	**0.473**	**<.0001**
COPD													**-11.312**	**0.816**	**<.0001**	**-10.741**	**0.808**	**<.0001**
Depression													**-19.113**	**0.559**	**<.0001**	**-18.221**	**0.554**	**<.0001**
Diabetes													**-5.202**	**0.477**	**<.0001**	**-4.809**	**0.470**	**<.0001**
Hypertension													**-4.055**	**0.287**	**<.0001**	**-3.860**	**0.285**	**<.0001**
**Number of diseases[j]**																		
One							**-7.298**	**0.271**	**<.0001**	**-6.865**	**0.268**	**<.0001**						
Two							**-13.426**	**0.450**	**<.0001**	**-12.608**	**0.447**	**<.0001**						
Three							**-25.177**	**1.201**	**<.0001**	**-23.673**	**1.174**	**<.0001**						
Four or more							**-29.101**	**2.731**	**<.0001**	**-27.799**	**2.643**	**<.0001**						
**Adjusted R^2**	0.1537			0.1708			0.1465			0.1620			0.1839			0.1962		
**RMSE**	17.17			17.00			17.25			17.09			16.86			16.74		
**N**	23,899			23,899			23,899			23,899			23,899			23,899		

Reference groups: [a]Men; [b]30–34 years; [c]Low educational level; [d]First income quintile (lowest); [e]No or occasionally smoking; [f]No risk consumption of alcohol; [g]150 minutes or more/week; [h]Sitting less than 10 hours per day; [ij]Not having the specific disease; [j]No disease. RSE: Robust Standard Error. Statistically significant estimates are shown in bold (<0.05)

Table S15 Ordinary Least Square (OLS) regression on mean EQ VAS score by Body Mass Index (BMI) groups, adjusted for sex, age, educational level and income, 30–104 years (n=23,899)

**Variable**	**EQ VAS score**					
	**Model 1**			**Model 2**		
	**Estimate**	**RSE**	**P-value**	**Estimate**	**RSE**	**P-value**
**Intercept**	**82.276**	**0.507**	**<.0001**	**75.719**	**0.665**	**<.0001**
**Sex**[a]						
Women	**-1.491**	**0.232**	**<.0001**	-0.328	0.245	0.1797
**Age group**[b]						
35-39	-0.199	0.663	0.7637	-0.800	0.664	0.2282
40-44	-0.384	0.644	0.5508	**-1.661**	**0.645**	**0.0100**
45-49	-0.393	0.628	0.5310	**-1.646**	**0.629**	**0.0088**
50-54	-0.925	0.632	0.1436	**-1.931**	**0.632**	**0.0023**
55-59	-0.305	0.634	0.6303	-1.234	0.632	0.0510
60-64	0.255	0.609	0.6757	-0.225	0.609	0.7119
65-69	1.116	0.583	0.0559	**1.488**	**0.584**	**0.0109**
70-74	-0.319	0.548	0.5601	**1.484**	**0.557**	**0.0077**
75-79	**-3.999**	**0.589**	**<.0001**	**-1.314**	**0.610**	**0.0311**
80-84	**-8.723**	**0.699**	**<.0001**	**-5.559**	**0.722**	**<.0001**
85-89	**-14.095**	**0.725**	**<.0001**	**-10.678**	**0.753**	**<.0001**
90-94	**-19.117**	**1.046**	**<.0001**	**-15.467**	**1.070**	**<.0001**
95-104	**-23.883**	**2.121**	**<.0001**	**-20.066**	**2.135**	**<.0001**
**Educational level**[c]						
Medium				**0.890**	**0.339**	**0.0086**
High				**1.644**	**0.361**	**<.0001**
Missing				*0.003*	*0.018*	*0.8843*
**Income** (individual) (thousand SEK)[d]						
Second quintile				**0.890**	**0.421**	**0.0347**
Third quintile				**4.267**	**0.409**	**<.0001**
Fourth quintile				**6.435**	**0.422**	**<.0001**
Fifth quintile				**7.364**	**0.436**	**<.0001**
Missing				***0.069***	***0.024***	***0.0035***
**BMI**[e]						
Underweight	**-6.655**	**1.210**	**<.0001**	**-6.186**	**1.193**	**<.0001**
Overweight	**-2.294**	**0.257**	**<.0001**	**-1.976**	**0.255**	**<.0001**
Obese class I	**-7.099**	**0.372**	**<.0001**	**-6.418**	**0.369**	**<.0001**
Obese class II	**-12.571**	**0.688**	**<.0001**	**-11.641**	**0.682**	**<.0001**
Obese class III	**-12.872**	**1.064**	**<.0001**	**-11.722**	**1.054**	**<.0001**
**Adjusted R^2**	0.1011			0.1211		
**RMSE**	17.70			17.50		
**N**	23,899			23,899		

Reference groups: [a]Men; [b]30–34 years; [c]Low educational level; [d]First income quintile (lowest); [e]Normal weight
RSE: Robust Standard Error. Statistically significant estimates are shown in bold (<0.05)

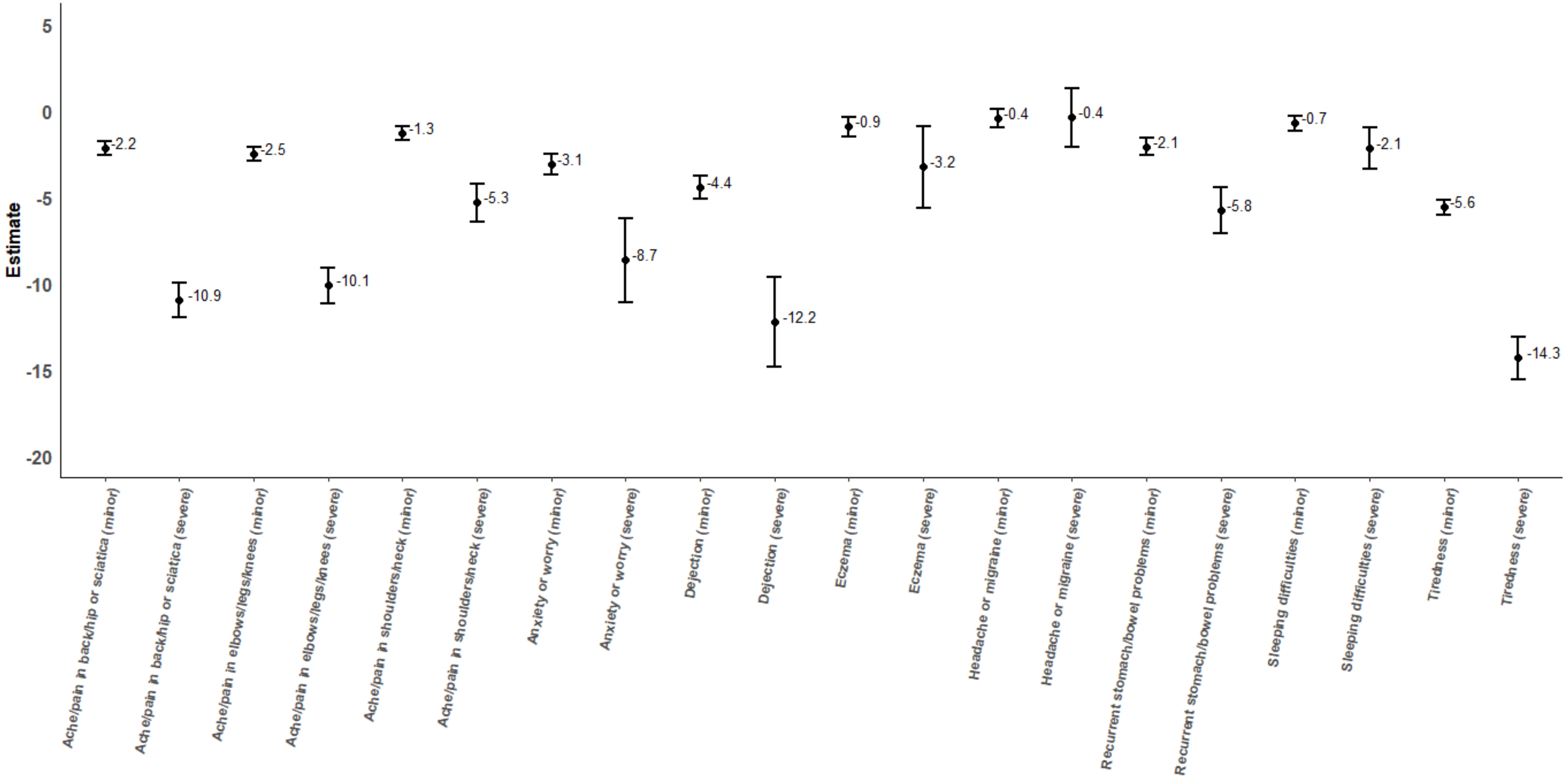

Figure S1 Ordinary Least Square (OLS) regression on mean EQ VAS score by self-reported conditions, adjusted for sex, age, educational level and income, estimates showing reduction in mean EQ VAS score with confidence intervals, 30–104 years (n=23,899)
